# Global transcriptome analysis reveals fungal disease responsive core gene regulatory landscape in tea

**DOI:** 10.1038/s41598-023-44163-x

**Published:** 2023-10-11

**Authors:** Anjan Hazra, Sanatan Ghosh, Sudipta Naskar, Piya Rahaman, Chitralekha Roy, Anirban Kundu, Rituparna Kundu Chaudhuri, Dipankar Chakraborti

**Affiliations:** 1 Department of Genetics, University of Calcutta, 35, Ballygunge Circular Road, Kolkata, 700019 India; 2 Plant Genomics and Bioinformatics Laboratory, P.G. Department of Botany, Ramakrishna Mission Vivekananda Centenary College (Autonomous), Rahara, Kolkata, 700118 India; 3 Department of Botany, Barasat Govt. College, 10, K.N.C. Road, Barasat, Kolkata, 700124 India

**Keywords:** Functional clustering, Gene regulatory networks, Biotic

## Abstract

Fungal infections are the inevitable limiting factor for productivity of tea. Transcriptome reprogramming recruits multiple regulatory pathways during pathogen infection. A comprehensive meta-analysis was performed utilizing previously reported, well-replicated transcriptomic datasets from seven fungal diseases of tea. The study identified a cumulative set of 18,517 differentially expressed genes (DEGs) in tea, implicated in several functional clusters, including the MAPK signaling pathway, transcriptional regulation, and the biosynthesis of phenylpropanoids. Gene set enrichment analyses under each pathogen stress elucidated that DEGs were involved in ethylene metabolism, secondary metabolism, receptor kinase activity, and various reactive oxygen species detoxification enzyme activities. Expressional fold change of combined datasets highlighting 2258 meta-DEGs shared a common transcriptomic response upon fungal stress in tea. Pervasive duplication events caused biotic stress-responsive core DEGs to appear in multiple copies throughout the tea genome. The co-expression network of meta-DEGs in multiple modules demonstrated the coordination of appropriate pathways, most of which involved cell wall organization. The functional coordination was controlled by a number of hub genes and miRNAs, leading to pathogenic resistance or susceptibility. This first-of-its-kind meta-analysis of host–pathogen interaction generated consensus candidate loci as molecular signatures, which can be associated with future resistance breeding programs in tea.

## Introduction

The most popular non-alcoholic beverage, tea, is known worldwide for its unique taste and health benefits. Shoots or tender leaves collected from tea shrubs (*Camellia sinensis* (L.) O. Kuntze) are used to make this second most consumed beverage after water^1,2^. Tea is a perennial crop that grows continuously in one place for long period of time (more than 50 years)^3^. As sessile organisms, tea plants are subject to a number of biotic and abiotic stresses that interfere with their normal growth and development^4–6^. As a result, there are significant losses in tea production across the globe. To manage pathogens and pests, farmers use fungicides or pesticides, which have led to an increase in the chemical load on processed tea as well as the acquirement of pesticide or fungicide tolerance in these biotic factors. To meet the demands of a growing population, it has become a major goal to develop high-yielding, pathogen- and pest-resistant crops using alternative methods. The use of various plant defense responses to harmful biotic challenges is essential for the healthy survival of tea plants in the same field^7^.

Plants have developed a complex defence system to cope with various pathogens. Plants can reconfigure cellular metabolism in response to a pathogen attack and induce an intricate, fine-tuned defence route. It is initiated by the recognition of the non-host organisms and defence against foreign molecules. When molecular patterns of pathogens are recognized by receptor molecules, a cascade of responses is triggered, culminating in the activation of the pattern-triggered immunity (PTI)^8^. If pathogens overcome the PTI defence by producing effectors in the host cell, plants can prevail against the pathogen by inducing a second array immune system, effector-triggered immunity (ETI)^9,10^. A tissue-delimited ETI induction often subdues pathogenic infection in distal tissues, activating the systemic acquired resistance (SAR)^11^. The cornerstones of plant immunity also involve the miRNA-mediated regulation pathways that maintain the gene expression to a level so that plant defence systems sustain longer^12^.

Transcriptome profiling research has been done in relation to tea fungal infections in a number of studies^13–19^. Those studies mostly report transcriptomic responses to specific pathogens; some do not implement the reference genome or use different versions of tea genomes. Recent chromosome-level genome assemblies for tea have made it possible to decode the precise genetic and physical positions of key genes and loci^20–22^. Unification of genomics and transcriptomic approaches is a prerequisite step toward translating crop improvement programs in tea^23,24^. Large numbers of pertinent transcriptome datasets are becoming publicly available due to the emergence of high-throughput next-generation sequencing technologies. Meta-analysis is an effective and powerful tool that can be used to integrate multiple studies and identify candidate genes of the plant stress response^25–28^. By amalgamating differentially expressed genes (DEG) therein transcriptomic data, it is easier to find core regulatory hubs for intricate biological processes^25,28,29^. In general, researchers may be able to identify common aspects of the interactions between the plant and various pathogens^30^. The earlier transcriptome profiling studies demonstrated how tea plants respond to fungal pathogenesis, and those researches were mainly focused on a single pathogen. However, such studies led to the generation of an ample volume of data required for a meta-analysis. In the present report, publicly available RNA-seq datasets have been incorporated from well-replicated studies of fungal infection in tea plants to comprehend the common transcriptional regulation. Functional enrichment and subjective interaction network analyses were performed to identify the key role played by the common DEGs in various metabolic pathways. Transcription factors (TFs) and candidate gene-targeting miRNA families were focused on elucidating the gene regulatory hubs. This consolidative study aimed to decipher useful insights into the regulatory mechanisms governed by tea plants when they encounter fungal pathogens and thereby modulation of tea quality due to these biotic challenges. The prioritized gene sets may be further considered for functional genomic strategies, such as locating useful molecular markers or modifying candidate genes to generate pathogen-resistant cultivars.

## Results

### Overview of the global datasets and transcriptome assembly

**Figure 1:**
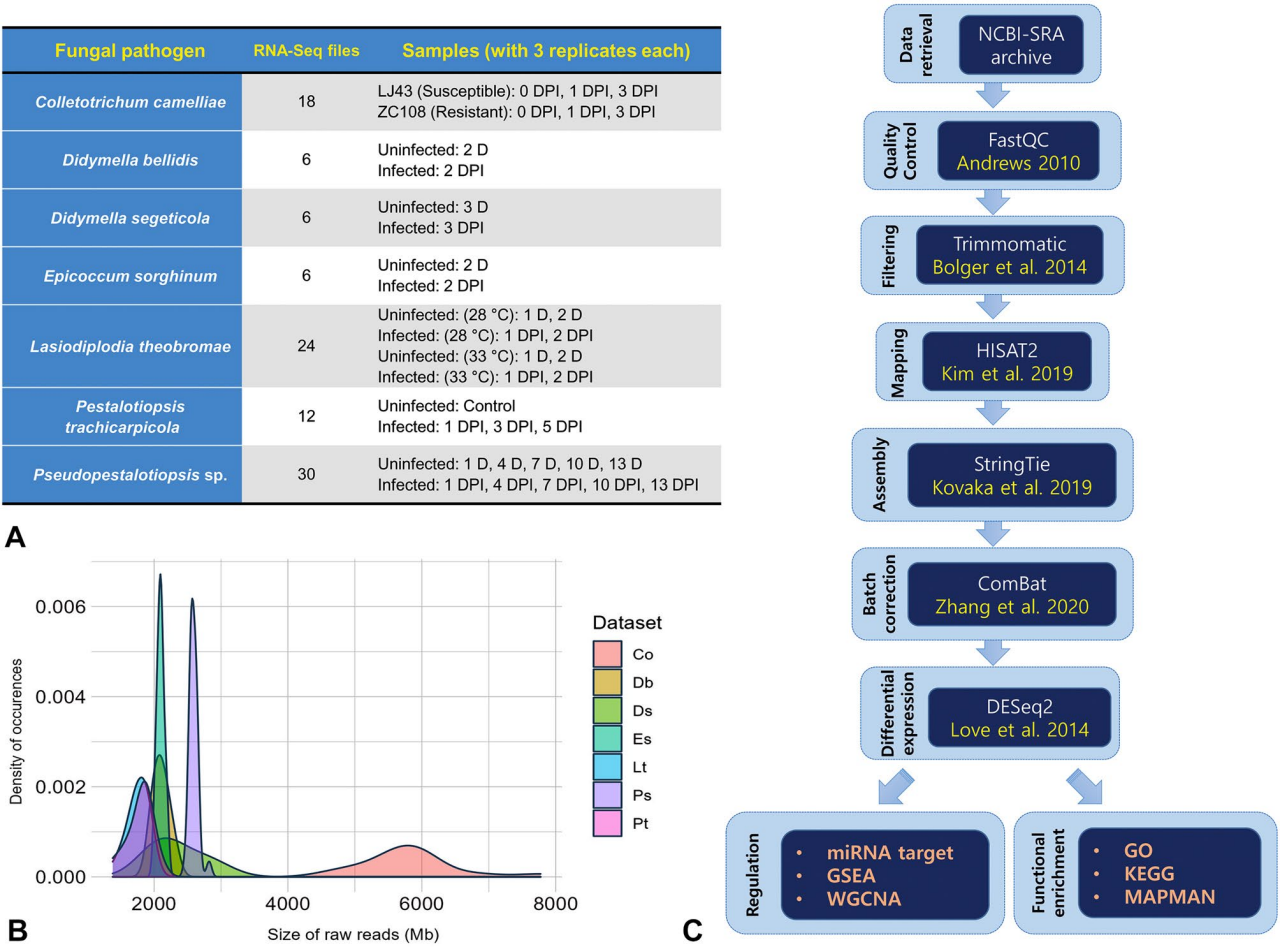
Overview of datasets and analysis workflow. Transcriptome datasets overview (**A**), density plot representing volumes of RNA-seq datasets (**B**), methodologies and tools implemented for transcriptome assembly and differential expression (**C**). Co—*Colletotrichum camelliae*, Db—*Didymella bellidis*, Ds—*Didymella segeticola*, Es—*Epicoccum sorghinum*, Lt—*Lasiodiplodia theobromae*, Pt—*Pestalotiopsis trachicarpicola* and Ps—*Pseudopestalotiopsis* sp. *D* days, *DPI* days post inoculation.

A total of 102 mRNA sequencing datasets encompassing seven fungal pathogen treatments, *i.e. Colletotrichum camelliae*^14^, *Didymella bellidis*, *Didymella segeticola*^18^, *Epicoccum sorghinum*^19^, *Lasiodiplodia theobromae*^16^, *Pestalotiopsis trachicarpicola*^13^ and *Pseudopestalotiopsis* sp.^17^ at multiple time intervals including uninfected or control samples were obtained from NCBI-SRA and categorized accordingly (Fig. 1A). The *C. camelliae* datasets, moreover, included transcriptomes from two different genotypes (LJ43 and ZC108) with contrasting resistance to the pathogen. Similarly, two identical experiments were set up in the case of *L. theobromae* in two different incubation temperatures, 28 °C and 33 °C. The raw read volumes of individual samples under seven pathogens were plotted in density, which depicts maximum mean read sizes in *C. camelliae* followed by a sharp distribution of *Epicoccum* and *Pseudopestalotiopsis* datasets (Fig. 1B). Raw reads of the remaining four pathogen-associated samples occurred in similar volumes. Nearly 16.4–63 million raw reads were acquired for all these samples (Supplementary Table S1), with > 99% retained as clean reads for the subsequent procedure (Fig. 1C). Following the reference-genome based transcriptome assembly, per sample gene counts and subsequent gene abundances were subjected to normalization for removal of undesirable variances and reduction of the batch effects among the datasets (Supplementary Tables S2–S5, Supplementary Fig. S1).

### Identification of differentially expressed genes

**Figure 2:**
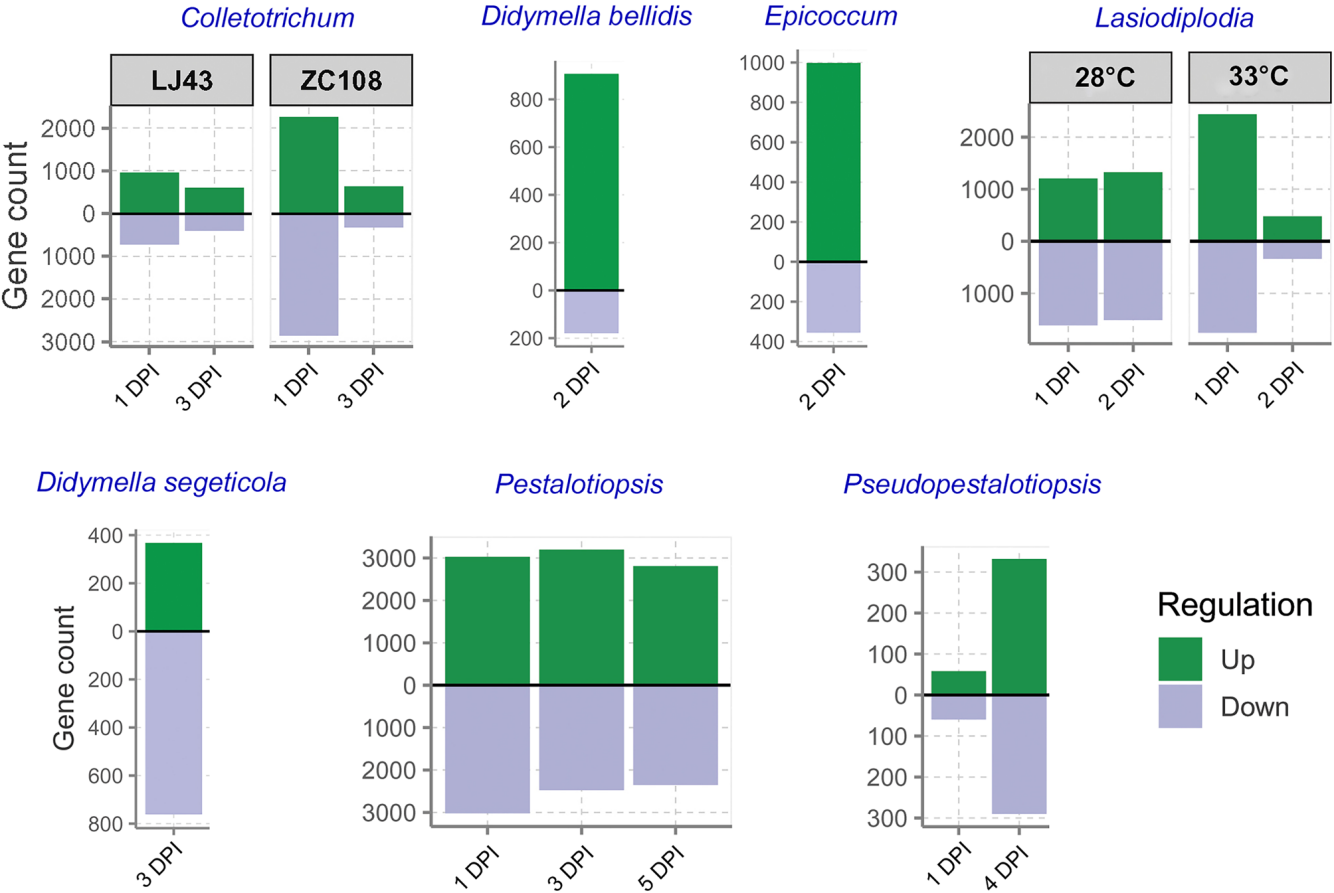
Diverging plots showing counts of differentially expressed genes in tea at various time points of seven different fungal infections. *DPI* days post-inoculation.

Using the DESeq2 statistical package^31^, only transcripts with a false discovery rate (FDR) adjusted p-value of less than 0.05 and log2(fold change) of more than 1 or less than − 1 (|log2foldchange| > 1) were considered significant. Different genotypes of the plants at multiple time points had differences in transcriptome profiles depending on how they responded to the pathogen (Fig. 2; Supplementary Table S2). A comparison of uninfected controls and *C. camelliae* treated plants at 1- and 3 days post-inoculation (DPI) revealed that 2262 and 632 genes had elevated expression, respectively, and 2852 and 315 genes had decreased expression, respectively, in the resistant genotype ZC108. In comparison, their susceptible counterpart, LJ43, at 1- and 3 DPI showed 958 and 599 up-regulated DEGs, respectively, and 718 and 390 down-regulated DEGs, respectively, in the experiments. Results of differential gene expression at single time points of *D. bellidis* (2 DPI), *D. segeticola* (3 DPI), *E. sorghinum* (2 DPI) pathogen inoculation in tea showed that a total of 1086 (907 up- and 179 down-regulated), 1129 (368 up- and 761 down-regulated), and 1353 (998 up- and 355 down-regulated) genes were manifested with significant alteration respectively. In response to *L. theobromae* inoculated at 28 °C, 1204 up- and 1612 down-regulated genes at 1 DPI, and 1325 up- and 1513 down-regulated genes at 2 DPI were observed. Simultaneously, at 33 °C and 1 DPI, 2438 genes went up and 1756 genes went down, and at 2 DPI, 480 genes went up and 334 genes went down. A total of 3029 up- and 3019 down-regulated genes were found in the 1 DPI of the gray blight-causing *P. trachicarpicola* pathogen experiment; the number gradually decreased up to 5 DPI. The transcriptome of a tea plant infected with a similar pathogen taxon, *Pseudopestalotiopsis sp.* led to the discovery of a small fraction of DEGs, with the highest occurrences (332 up- and 289 down-regulated genes) at 4 DPI in comparison to 58 up- and 59 down-regulated genes at 1 DPI. Due to the low number of DEGs detected in the later days of this dataset and to maintain parity with other pathogen-related datasets of early time point responses, the 7–13 DPI samples were excluded from further analysis. In the upset diagram (Supplementary Fig. S2), DEGs observed in various pathogen treatments were compared to find unique and shared ones. Altogether, 18,517 (36.6%) DEGs could be detected among the 50,525 predicted genes in the reference tea genome that are modulated following the onset of any of the seven studied fungal infections (Supplementary Table S6).

### Pathways involved in response to various fungal stress

**Figure 3:**
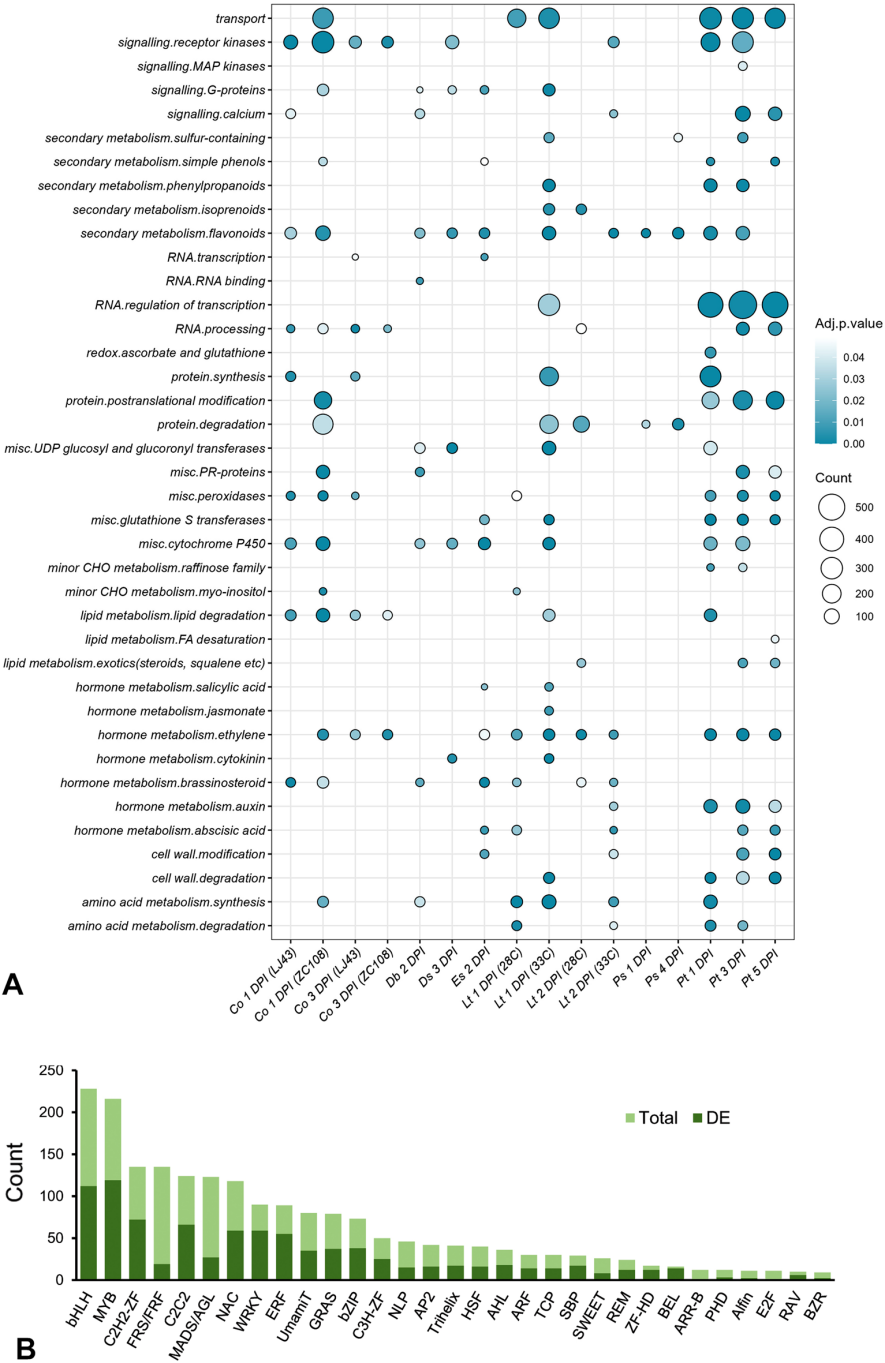
Summary of DEGs identified from various molecular functions associated with biotic stress. Pathway enrichment with significant threshold (Adj.p.value < 0.05) for identified DEGs under different conditions performed in MAPMAN platform (**A**), proportions of transcription factors with substantial alteration to pathogen treatment; light and dark green represent total and differentially expressed (DE) genes, respectively (**B**). *DPI* days post inoculation.

**Figure 4:**
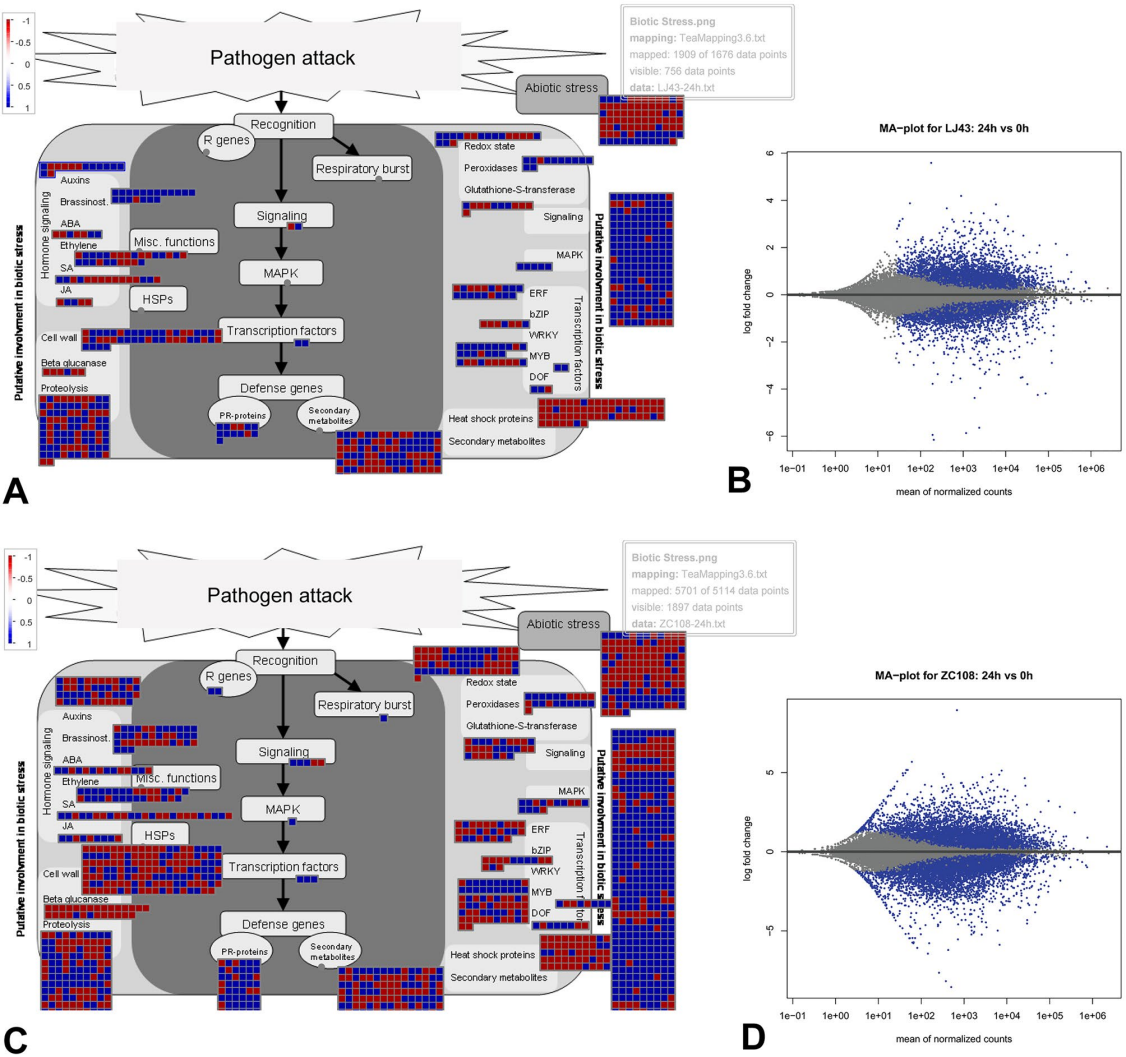
Identified DEGs and their mapping to biotic stress pathways determined by MAPMAN and MA plots. Diagrammatic representation of the biotic stress associated DEGs involved in responses to *Colletotrichum* using MAPMAN bin (**A**) and MA plot (**B**) in susceptible and MAPMAN bin (**C**) and MA plot (**D**) in resistant genotype. The gradient of color scale representing the log fold ratios is related to the magnitude of gene expression.

To comprehend how the fungal infection affected metabolic pathways in tea at various time points, the resultant DEGs were clustered into various functional categories using enrichment in MapMan^32^ bins (Fig. 3, Supplementary Table S7). When challenged with *C. camelliae*, many genes encoding multiple components of brassinosteroid synthesis, protein synthesis, and RNA processing were significantly enriched in the susceptible cultivar LJ43 (Figs. 3A, 4A,B). On the other hand, genes involved in ethylene metabolism, Myo-inositol synthesis, posttranslational modification, flavonoid metabolism, various transport mechanisms, and the synthesis of PR-proteins were prominently active in the resistant genotype ZC108, particularly early point of infection (Figs. 3A, 4C,D). Comparison of expressional fold changes of the secondary metabolism pathway genes between the genotypes revealed that most flavonoid biosynthesis genes were down-regulated upon fungal elicitation. In contrast, the terpenoid biosynthetic genes were mainly up-regulated (Supplementary Fig. S3). Consistent with the findings, two negative regulators of essential phenylpropanoid pathway genes, CSS0001528 (KFB-CHS) and CSS0009976 (KFB-PAL), were observed to be elevated congruently. Notably, 2-hydroxyisoflavanone dehydratases (CSS0003974 and CSS0030793) and flavonol 3-O-glycosyltransferases (CSS0009012, CSS0015218, and CSS0033573), those involved in the conversion of flavonoid into isoflavones and glycosylated derivatives, were up-regulated in both the backgrounds. The widely known R-genes and MAP kinases (MAPKs) that play important roles in defense signaling were poorly represented in the susceptible plant. They showed distinctly higher induction in the resistant genotype (Fig. 4C). Other over-represented categories in the resistant counterpart include transcription factors, hormone signaling, and heat shock proteins. The processes of lipid degradation, receptor kinases, cytochrome P450, and peroxidase activities were impacted in both backgrounds, as observed through the allotment of MapMan bins in respective categories. At 28 °C, eliciting tea leaf spot-causing *L. theobromae* resulted in an increased modulation of transcripts related to amino acid metabolism and various transport mechanisms, among all other components of the biotic stress-mediated pathway. The scenario changed, however, when altered metabolic processes at 33 °C were visualized, which is known to significantly accelerate hyphae cell growth and affect the host–pathogen interaction mechanism. A more remarkable alteration in cell wall degradation, metabolism of cytokinin, jasmonic acid (JA) and salicylic acid (SA), secondary metabolic pathways for flavonoids, isoprenoids, and phenylpropanoids production, G-proteins, cytochrome P450, and glutathione S transferases were observed in the same subset. When the tea plant was exposed to *P. trachicarpicola*, another phytopathogenic fungus, all of the pathways mentioned above were significantly enriched during the infection process.

Nevertheless, important findings regarding auxin metabolism and transcriptional regulation in this specific host–pathogen interaction were of pertinent interest. Despite a comparatively smaller number of DEGs identified in *Pseudopestalotiopsis *sp. inoculated datasets, protein degradation, and flavonoid metabolism were designated as important ones with prodigious statistical support among other bins. DEGs observed at single time points after inoculation of *D. bellidis*, *D. segeticola*, *E. sorghinum* resulted in a similar trend with induction of ethylene metabolism, antioxidant and detoxification, protein modification and degradation, transcription factor and receptor kinase-related genes (Fig. 3A). The Kyoto Encyclopedia of Genes and Genomes (KEGG)^33^ pathway-based gene set enrichment analysis (GSEA) represented using ridge plots showed significant modulation of MAPK signaling, phenylpropanoid biosynthesis, and plant-pathogen interaction pathways constant across all pathogen stresses, in addition to multifaced unique layer of changes upon individual fungal responses (Supplementary Fig. S4-S9).

### Involvement of transcription factors

Plants' ability to respond to stress depends on the regulation of gene expression by transcription factors (TFs). Since transcription factors were highly enriched among the MAPMAN bins, a total of 1982 TF genes representing 31 important TF families were further examined. Amongst these, the bHLH, MYB, C2H2-ZF, FRS/FRF, C2C2, MADS/AGL, NAC, WRKY, ERF, UmamiT, GRAS, bZIP and C3H-ZF families were predominant in terms of the number of TF genes (Fig. 3B). Expression of the MYB, bHLH, C2H2-ZF, C2C2, WRKY, and NAC families was most differentially regulated upon fungal infections in tea. When the proportion of differentially expressed members among each TF family was considered, ZF-HD was highest with 70.6% differentially up- or down-regulated members under fungal infection followed by WRKY (65.5%), ERF (61.8%), RAV (60%), SBP (58.6%), MYB (55%), C2C2 (53.2%), C2H2-ZF (53.3%) and bZIP (52%). Further information about the individual transcription factors encoding genes that were differentially regulated in all the studied conditions under individual stress factors can be found in Supplementary Table S6.

### Differential regulation of plant immune-related signal transduction genes

**Figure 5:**
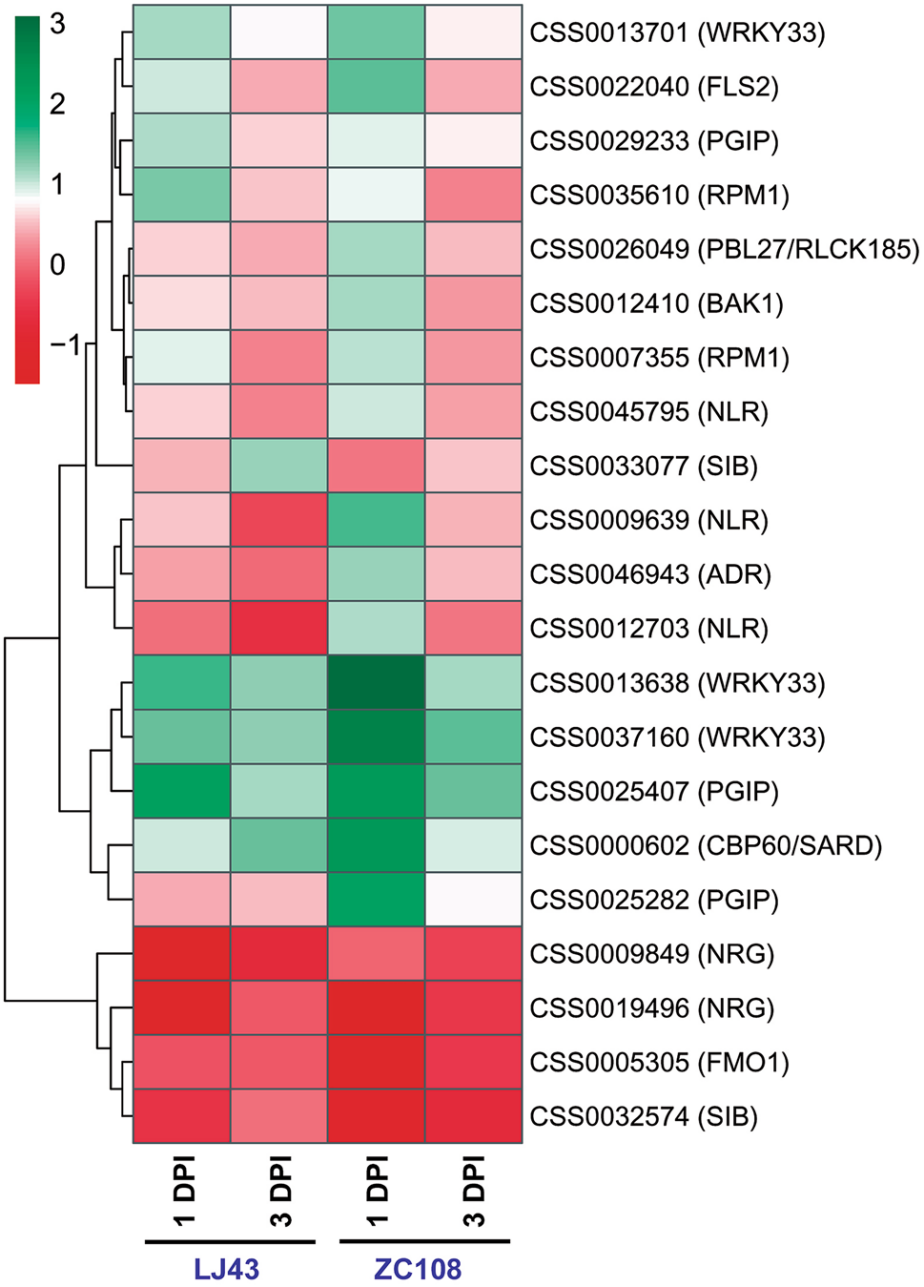
Heatmap depicting expressional fold change of pathogen-associated signal transduction genes between susceptible and resistant genotypes. *DPI* days post inoculation.

Expressional dynamics of external stimuli (pathogen) response genes in tea plants were compared between susceptible and resistant backgrounds while challenged with *C. camelliae* (Fig. 5). Members of the pathogen polygalacturonase inhibitor (PGIP, CSS0025282) and pattern-triggered immunity, BAK1 (CSS0012410) and PBL27/RLCK185 (CSS0026049), were specifically up-regulated in the resistant genotype. A similar trend was also observed in several components of effector-triggered immunity (ETI), such as NLR (CSS0009639, CSS0012703, and CSS0045795), ADR (CSS0046943), and RPM1 (CSS0007355). TNL-mediated effector-triggered immunity regulatory proteins, NRG (CSS0009849 and CSS0019496), and systemic acquired resistance regulatory proteins, FMO1 (CSS0005305), were down-regulated in both genotypes. Another systemic acquired resistance regulatory protein, CBP60/SARD (CSS0000602), and plant immunity-related WRKY33 transcription factors (CSS0013638 and CSS0037160) were up-regulated in both backgrounds but with higher magnitudes in the resistant one.

### Modulation of genes related to flavonoid biosynthesis

**Figure 6:**
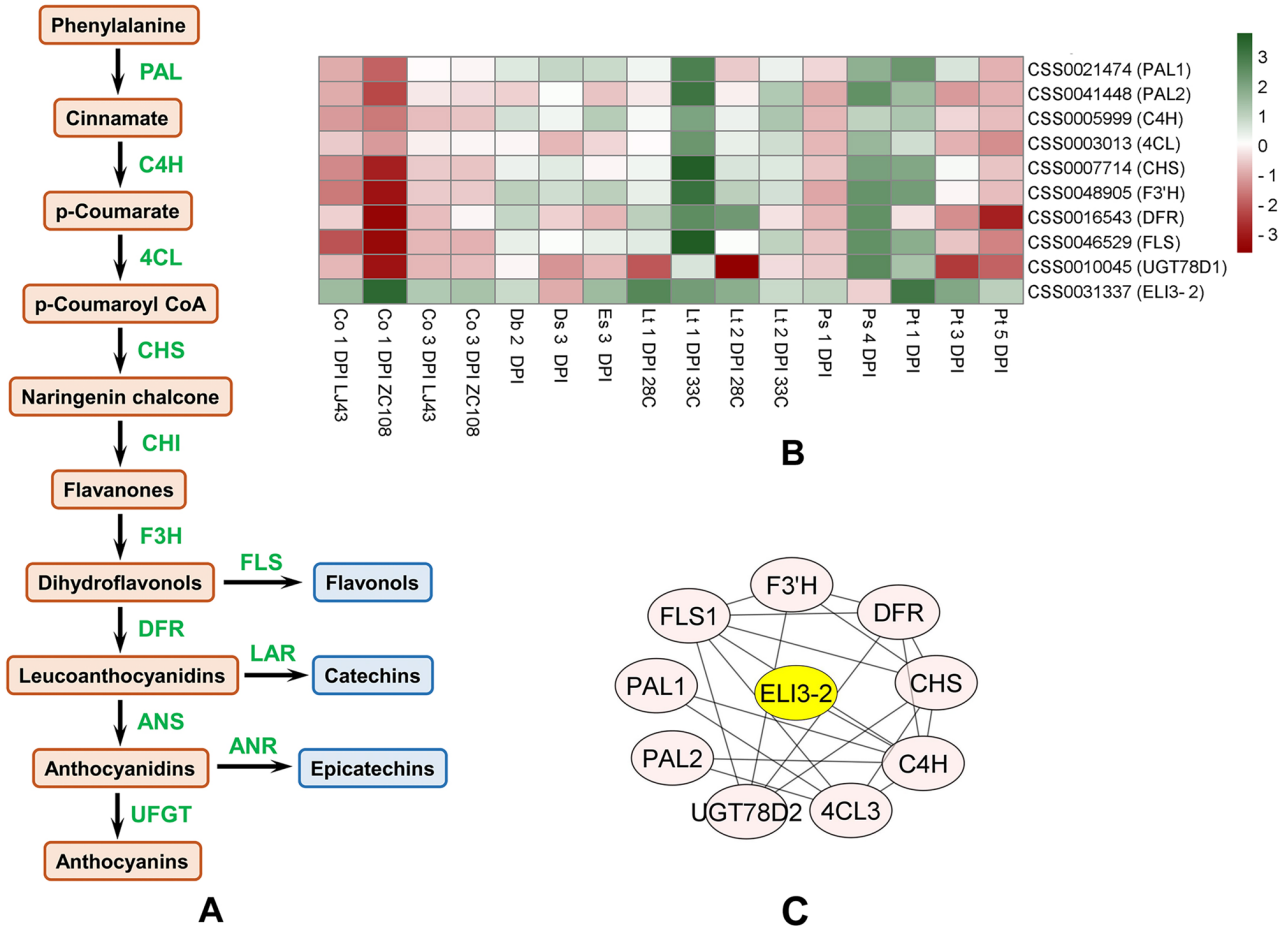
DEGs related to tea flavonols metabolism. Diagram of the section of phenylpropanoid pathway leading to the production of primary flavonol compounds in tea (**A**), expressional fold change of corresponding genes in the examined condition (**B**), significant interaction modules among the pathway genes derived upon fungal stress (**C**). *ANR* anthocyanidin reductase, *ANS* anthocyanidin synthase, *4CL* 4-coumarate:CoA ligase, *C4H* cinnamate 4-hydroxylase, *CHI* chalcone isomerase, *CHIL* chalcone isomerase-like, *CHS* chalcone synthase, *DFR* dihydroflavonol 4-reductase, *F3GT* flavonoid 3-O-glucosyltransferase, *F3H* flavanone 3-hydroxylase, *FLS* flavonol synthase, *LAR* leucoanthocyanidin reductase, *PAL* phenylalanine ammonia-lyase, *UFGT* UDP-glucose:flavonoid 3-O-glucosyltransferase. *DPI* days post inoculation.

The MapMan enrichment and KEGG pathway-based GSEA indicated that fungal pathogenesis strongly influences tea flavonoid metabolism. The pool of differentially expressed genes discovered in this study included essential genes for flavonoid biosynthesis (Fig. 6A,B, Supplementary Table S6). Protein–protein interaction (PPI) networks of the shared differentially expressed pool of genes were analyzed in STRING^34^, based on the strong confidence (> 0.9) of interaction among the identified genes, to understand the regulatory interaction of this pathway. As a result, an interaction cluster was discovered that included the genes encoding required enzymes for the flavonoid biosynthesis pathway and one additional member, CSS0031337 (Fig. 6C, Supplementary Table S8). Those were comprised of flavonol synthase (FLS; CSS0046529), flavonoid 3′-hydroxylase (F3'H; CSS0048905), flavonol-3-O-rhamnosyltransferase (UGT78D1; CSS0010045), dihydroflavonol 4-reductase (DFR; CSS0016543), chalcone synthase (CHS; CSS0007714), 4-coumarate:coenzyme A ligase (4CL; CSS0003013), cinnamate-4-hydroxylase (C4H; CSS0005999) and phenylalanine ammonia-lyase (PAL; CSS0021474 and CSS0041448) (Fig. 6B). Interestingly, the sequence of CSS0031337, obtained in the same module, was observed, to be as a mannitol dehydrogenase. However, when investigated with this sequence in The Arabidopsis Information Resource (TAIR) database, it revealed homology with AT4G37990, the elicitor-activated gene 3–2 (ELI3-2). ELI3-2 encodes aromatic alcohol: NADP + oxidoreductase, the expression of which increases in response to phytopathogenic bacteria. This enzyme does not have mannitol dehydrogenase activity despite the similarity with their gene sequence. In the current analyses, the majority of tea transcriptomes exposed to fungal pathogens showed elevated expression of this particular gene (Fig. 6B). Coordinated fold changes of flavonol biosynthesis pathway-related genes occurred in the pathogen-treated datasets during the onset of infection. At 1 DPI, this cluster was down-regulated in the anthracnose-resistant genotype ZC108, while ELI3-2 had a strong upregulation in the same system. Expressional magnitudes of the other two pathways changed diversely under various conditions.

### Identification of meta-DEGs and functional enrichment

**Figure 7:**
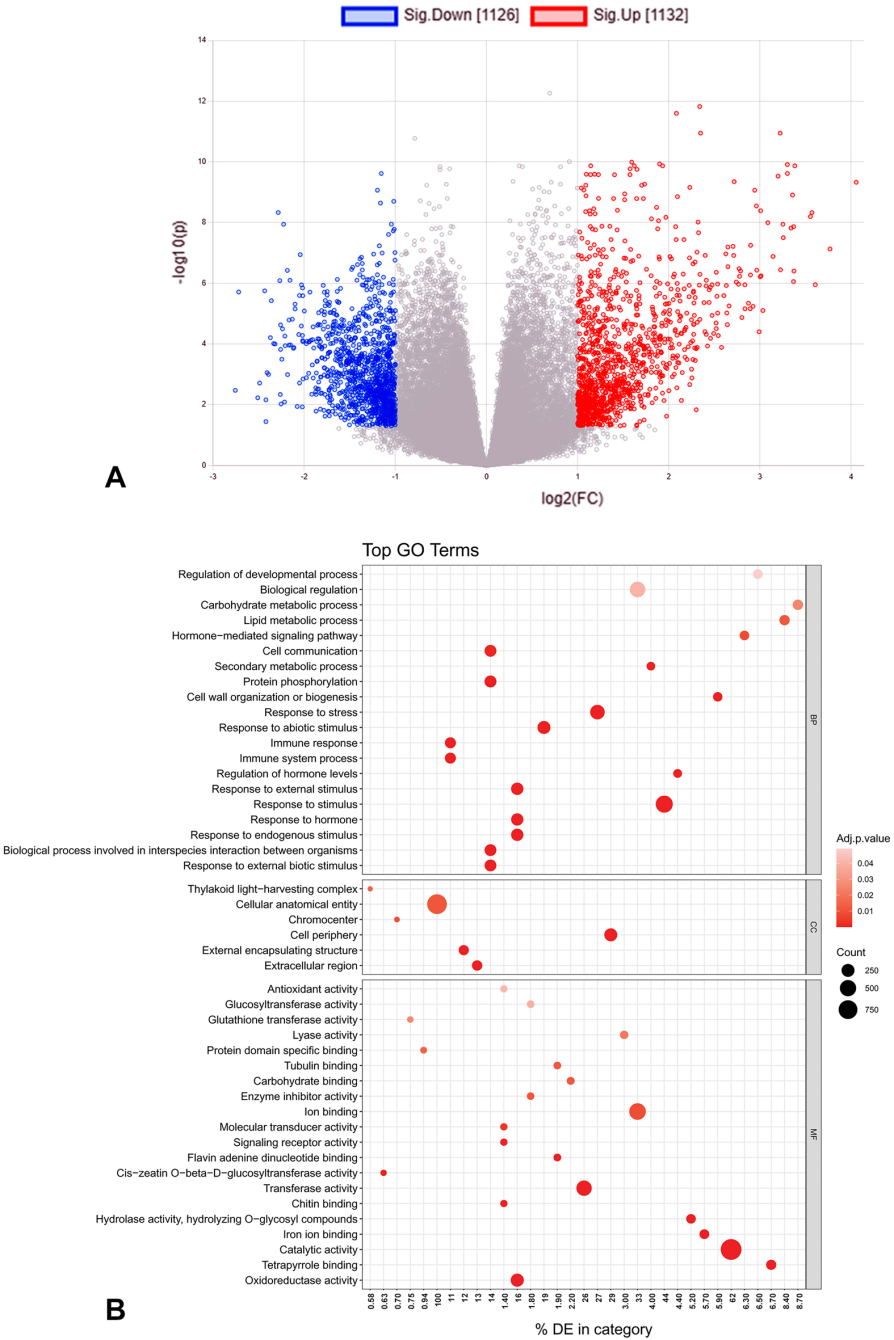
Meta-DEGs identified from global transcriptome datasets. Volcano plot indicating significant up- and down-regulated genes in overall conditions (**A**), GO terms overrepresented in biological process, cellular component, and molecular function (**B**). A lower p-value indicates a higher degree of enrichment, as shown by a more significant enrichment coefficient.

**Figure 8:**
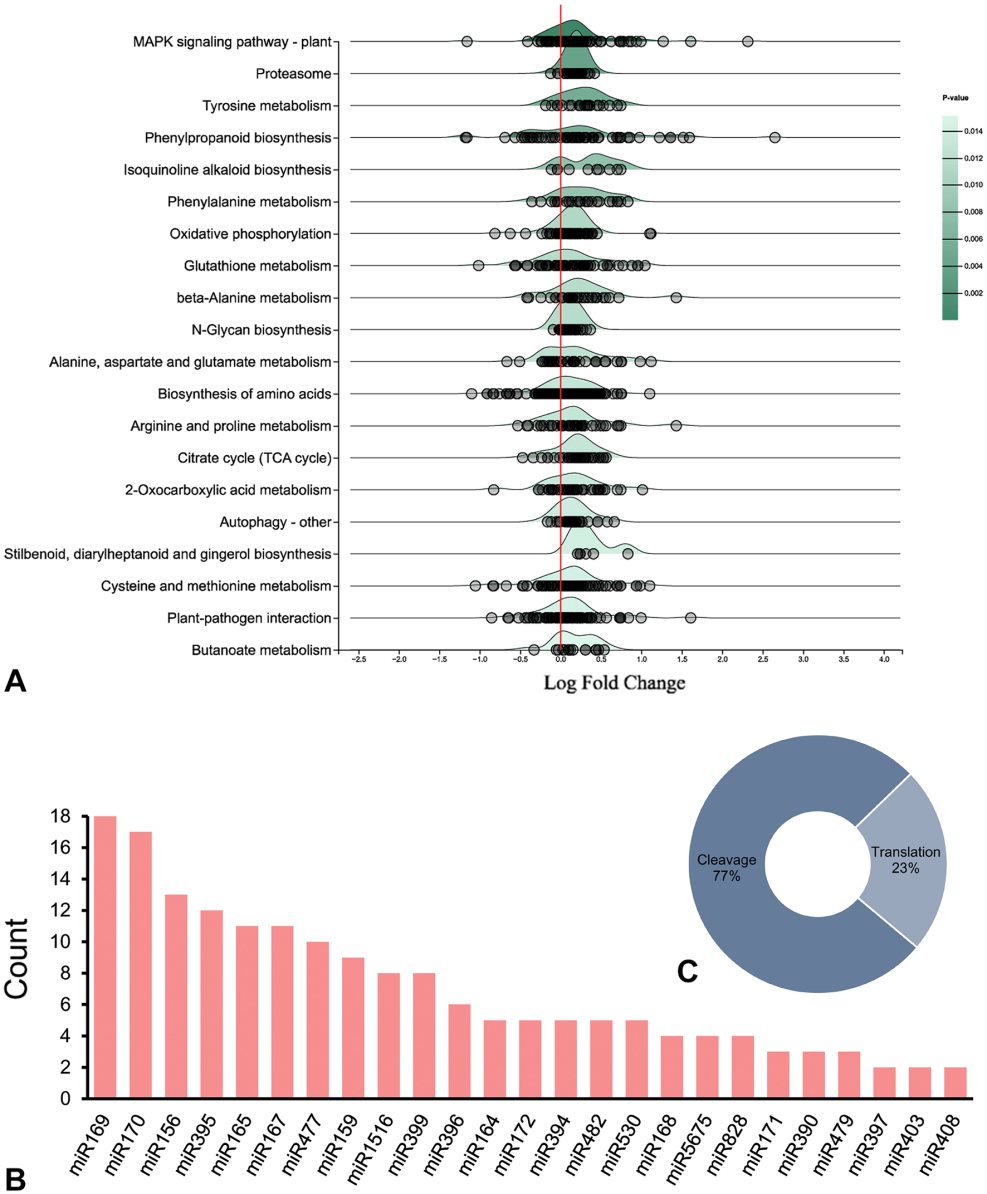
Summary of gene set enrichment analysis with meta-DEGs and their miRNA target prediction. Density ridgeline plots displaying enrichment of DEGs in corresponding KEGG^33^ pathways (**A**), major miRNA families targeting the Meta-DEGs (**B**), mode of miRNA mediated regulation (**C**).

In a meta-analysis, the fold change of a gene is calculated as the average fold changes for that gene across all studies. By comparing the infected and control groups while keeping individual conditions as a blocking factor, 2258 genes had differential expressions with significant cutoffs (|log2FC| > 1, p < 0.05). Of these meta-DEGs, 1132 genes were up-regulated, and 1126 were down-regulated (Fig. 7A, Supplementary Table S9). Gene ontology (GO) enrichment of the meta-DEGs with three GO terms: biological process (BP), cellular component (CC), and molecular function (MF) led to the identification of the major functional groups operating upon fungal pathogens (Fig. 7B; Supplementary Table S10). The significant GO terms related to the cellular anatomical entity, cell periphery, and extracellular region were discovered to be significantly enriched among the cellular components. At the same time, the thylakoid light-harvesting complex was only marginally represented. In terms of biological processes, "response to stimulus" (GO:0050896), "response to stress" (GO:0006950), "response to endogenous stimulus" (GO:0009719), "response to hormone" (GO:0009725), "response to external stimulus" (GO:0009605), "response to external biotic stimulus" (GO:0043207), "biological process involved in interspecies interaction between organisms" (GO:0044419), "protein phosphorylation" (GO:0006468), "cell communication" (GO:0007154), "immune response" (GO:0006955) and their subpart processes were the most commonly represented categories. There was also a notable enrichment in the molecular function categories of "catalytic activity" (GO:0003824), "ion binding" (GO:0043167), "transferase activity" (GO:0016740), "oxidoreductase activity" (GO:0016491), "tetrapyrrole binding" (GO:0046906), "iron ion binding" (GO:0005506), "hydrolase activity, hydrolyzing O-glycosyl compounds" (GO:0004553), "lyase activity" (GO:0016829), "carbohydrate binding" (GO:0030246), "flavin adenine dinucleotide binding" (GO:0050660), "tubulin binding" (GO:0015631), "enzyme inhibitor activity" (GO:0004857), "glucosyltransferase activity" (GO:0046527), "chitin-binding" (GO:0008061), "signaling receptor activity" (GO:0038023), "molecular transducer activity" (GO:0060089), "antioxidant activity" (GO:0016209), whose constituents may be crucial in determining the host's defensive response. GSEA enrichment analyses were performed using meta-DEGs with assigned KEGG orthologous numbers to various metabolic pathways. The highly represented groups included "MAPK signaling pathway", "Proteasome", "Phenylpropanoid biosynthesis", "Oxidative phosphorylation", "Biosynthesis of amino acids", "N-Glycan biosynthesis" and "Plant pathogen interaction" (Fig. 8A).

### miRNAs targeting meta-DEGs

MicroRNAs (miRNAs) are a group of non-coding small RNA molecules that play essential roles in the plant immune response. We used the psRNATarget^35^ tool to find miRNAs possibly linked to the regulation of the DEGs cumulatively identified in this study (Supplementary Table S11). The analyses revealed 128 transcripts as potential targets of one or more miRNAs. There were 302 different miRNAs found to target the DEGs. The most target transcripts were found in the miR169, miR170, miR156, miR395, miR165, miR167, miR477 families, in that order (Fig. 8B). The observed miRNAs repressed target gene expression primarily through transcript cleavage and a smaller fraction through translational repression (Fig. 8C). The top statistically significant miRNA target transcripts predicted by psRNATarget were checked for degradome-validated evidence in the TarDB portal^36^. It revealed that miR170 and miR397 families target GRAS transcription factors and laccases, respectively, as prophesied in our analyses. Notably, the CC-NLR-type effector receptor (RPM1), a component of the ETI network facilitating plant immune response, was found to be targeted by Csi-miRN5258. However, Csi-miRN5195, Csi-miRN5290, Csi-miRN5300, and Csi-miRN5307 were directed to cleave several flavonoid biosynthetic pathway genes. When expressional fold change of some miRNA candidates compared with their differentially expressed targets, some of the miRNA up/down-regulation incidences were co-occurring with corresponding down/up-regulated target transcripts (Supplementary Table S12).

### Chromosomal mapping and gene duplication analysis

**Figure 9:**
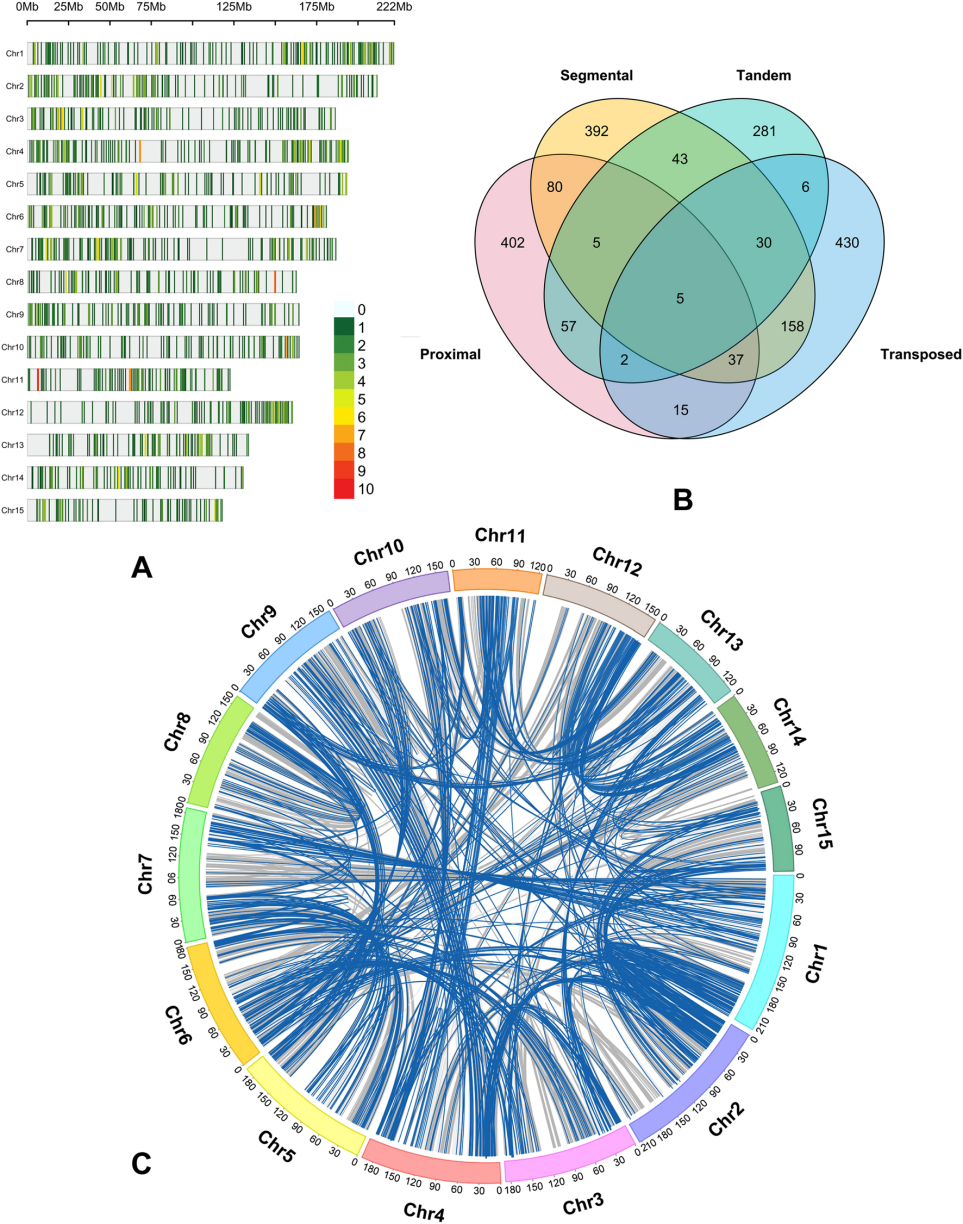
Chromosomal mapping and duplication analysis of the fungal infection-responsive meta-DEGs observed in tea. Physical map of the chromosomal position of genes (**A**), comparison of genes undergone various types of duplication (**B**), synteny analysis showing identified meta-DEGs along 15 chromosomes of tea plant (**C**).

A total of 1954 genes were annotated onto 15 chromosomes on the tea genome. The physical map was constructed by plotting the genes based on their chromosomal location (Fig. 9A). The majority of them, 182 genes, are located on chromosome 4, followed by 173 genes on chromosome 2, 172 genes each on chromosomes 1, 160 genes on chromosome 7. Particular segments of chromosomes were specifically enriched with multiple DEG loci. The duplication analysis of the meta-DEGs within tea genomes suggested most of them are duplicated genes. It was found that 603, 751, 429, and 683 genes underwent proximal, segmental, tandem, and transposed types of duplication, respectively (Fig. 9B). Several genes were predicted to be consequences of multiple duplication types. Collinearity analysis was performed to investigate further the expansion of identified gene sets (Fig. 9C). Interestingly, the results revealed that duplicated paralogous genes shared many syntenic gene pairs, signifying the fact that biotic stress-responsive genes or gene families underwent genome-wide expansion in tea during evolution.

### Identification of co-expressed modules and hub genes

**Figure 10:**
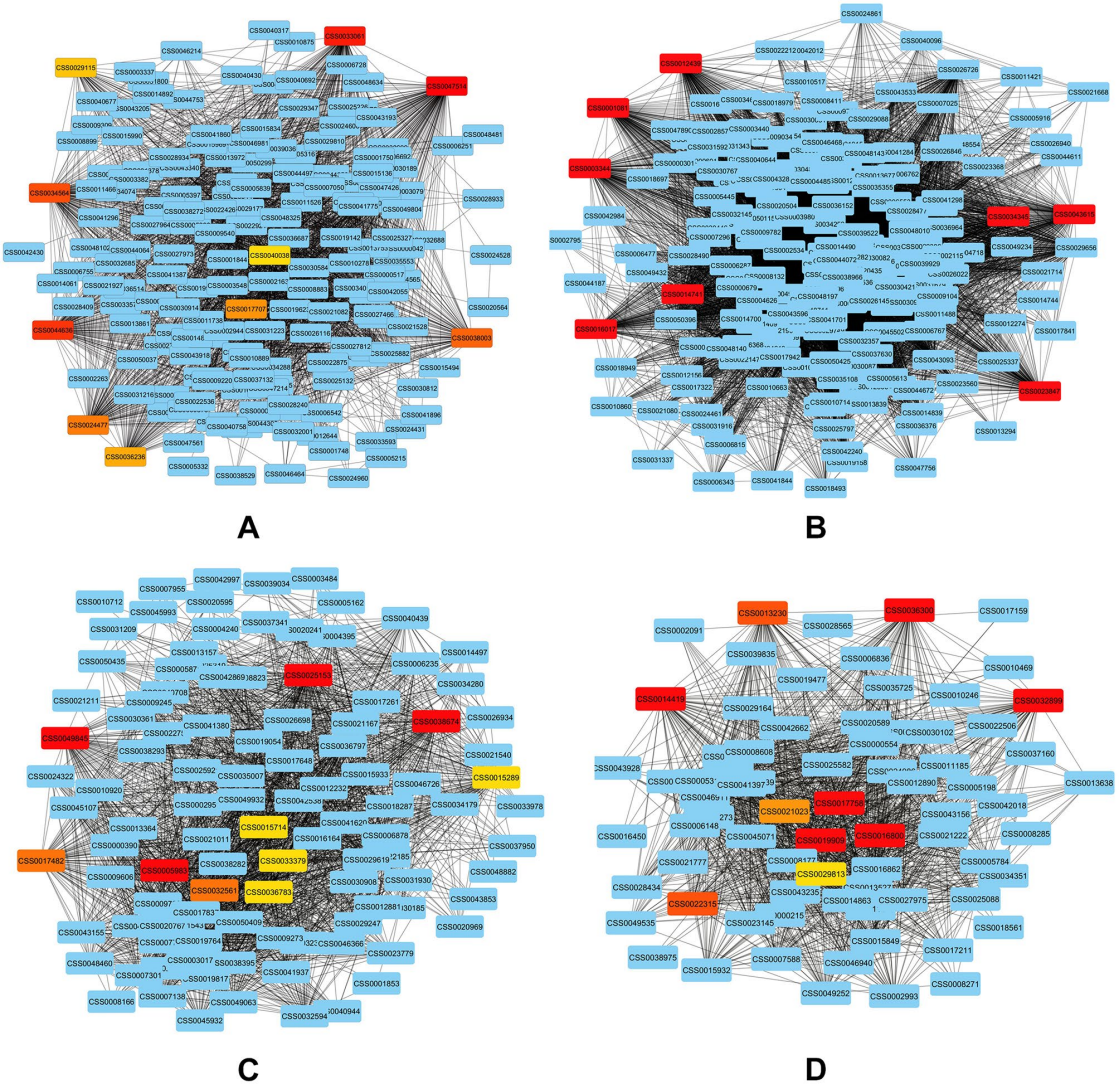
Co-expressed genes with edge weight > 0.10 in top 4 overrepresented modules. Module 1 (**A**), 2 (**B**), 3 (**C**), 4 (**D**). Each node in the network represents a gene. The genes that are co-expressed are linked by a grey line. Hub genes identified by cytoHubba are indicated in red with a gradient according to rank.

In order to find the genes that are highly correlated among all meta-genes across various pathogenic fungus treatments to tea, a Weighted Correlation Network Analysis (WGCNA) was carried out (Supplementary Table S13, Supplementary Fig. S10). A total of 832 genes were identified to build six modules with consistent expression patterns across samples. Essential hub genes displaying maximum network interactions were highlighted (Fig. 10A–D). Differential expression of this group of genes occurred during pathogen infection and encoded proteins that are either directly or indirectly regulated by pathogens. The roles played by important hub genes during pathogenic interactions could be functionally distributed into several categories, such as cell wall organization (alpha-class expansin, cutin synthase, pectate lyase, etc.), RNA biosynthesis (ERF, bZIP, MYB, bHLH, C2H2-ZF transcription factors), oxidoreductases, phytohormone action, protein homeostasis and secondary metabolism (Table 1). Among the group members, a type-II flavone synthase gene (CSS0007273) was the target of a specific miRNA (Csi-miRN5307).

**Table 1:** Identified hub genes from various co-expression modules.

Class	Gene families	Gene IDs
Cell wall organisation	Alpha-class expansin	CSS0027358, CSS0044072, CSS0005371, CSS0038939, CSS0005945, CSS0043596
	Fasciclin-type arabinogalactan protein (FLA)	CSS0034345
	Cellulose modification enzyme (class-B endo-1)	CSS0040022
	Cuticular lipid formation enzyme (fatty acyl in-chain hydroxylase)	CSS0031915, CSS0002756
	Fatty acyl omega-hydroxylase (CYP86A)	CSS0048711, CSS0033566
	Cutin synthase (CD)	CSS0004431, CSS0026875, CSS0014773, CSS0050396
	Xylan biosynthesis enzyme (galacturonosyltransferase)	CSS0013171, CSS0014419, CSS0013230
	Xyloglucan modification factor	CSS0010238
	Pectin methylesterase	CSS0001081
	Pectate lyase	CSS0018895
Chromatin organisation	Histone (H2A)	CSS0022583
	Histone (H3)	CSS0044204, CSS0025239, CSS0009943
Oxidoreductases	Acting on CH-OH group of donor	CSS0049845, CSS0005983, CSS0025153, CSS0032561
	Acting on diphenol or related substance as donor	CSS0036236
	Acting on paired donor with incorporation or reduction of molecular oxygen	CSS0038003, CSS0017707
Hydrolases	Glycosylase	CSS0014741, CSS0012190
Lipid metabolism	Glycerol-3-phosphate acyltransferase (GPAT4-8)	CSS0016066
Multi-process regulation	Small GTPase (ROP)	CSS0011478
Photosynthesis	LHC-I complex component LHCa4	CSS0009796
	Photosystem II assembly regulatory protein (Psb28)	CSS0012566
	pH-sensor protein (PsbS)	CSS0039743, CSS0029272
	Glycolate oxidase	CSS0033061
Phytohormone action	GASA-precursor polypeptide	CSS0043093
	RALF/RALFL-precursor polypeptide	CSS0043615
	PIP/PIPL-precursor polypeptide	CSS0015714
Protein homeostasis	A1-class protease (Pepsin)	CSS0029749
	M24-class methionyl aminopeptidase (MAP1)	CSS0044636
	M10-class metalloprotease (Matrixin)	CSS0032899
	Kunitz protease inhibitor	CSS0038674
	Ubiquitin-ligase E3 activities (RING-domain E3)	CSS0016800, CSS0017758
	U-Box E3 ligase activities (group-III ligase)	CSS0036300
Redox homeostasis	Glutathione S-transferase	CSS0015289, CSS0036783
	Atypical 2-Cys peroxiredoxin (PrxQ)	CSS0005573
RNA biosynthesis (transcriptional regulation)	Transcription factor (ERF)	CSS0030082
	Transcription factor (bZIP)	CSS0048197, CSS0020026
	Transcription factor (MYB)	CSS0024477
	Transcription factor (bHLH)	CSS0020435
	Transcription factor (C2H2-ZF)	CSS0021023
Secondary metabolism	Type-II flavone synthase	CSS0007273

## Discussion

Field-grown plants experience numerous biotic stresses and respond by changing their transcriptome in a coordinated way. Although these changes depend on the particular stress encountered, prior research has shown that all stresses have a similar core response^27,29^. By comparing similarities between related, independent studies, it is possible to find the common genes most strongly linked to the trait under investigation and shortlist them for functional analysis^37^. Many signalling molecules play a role in the plant's reaction to stress, including plant hormones and secondary metabolites, which are important regulators of genetic switches and cellular adjustments in a stressful environment^38^. The current study, a first-ever report, sought to comprehend the overall transcriptional regulation governed by tea under multiple fungal infections and their potential links to metabolic pathways involved in tea quality. The core set of genetic components obtained from the present study can further be utilized as markers for the future research on pathogenic resistance or susceptibility in tea.

When the plant recognises a pathogen's attack, it responds by up-regulating the synthesis of various proteins. Antibiotic proteins like PR proteins are among those. PR proteins have antifungal properties like facilitating fungal cell wall hydrolysis and are frequently recognized as indicative markers for systemic-acquired resistance (SAR)^39^. SA, ethylene, and JA signaling pathways can up-regulate PR genes^40,41^. The present study highlights that simultaneous upregulation of ethylene-responsive genes and the PR protein family occurred in the anthracnose-resistant tea genotype, that too at the early infection stage. These can imply their role in activating the defense system upon recognizing the pathogen, creating an incompatible environment to cause the disease. PTI responses work by identifying conserved pathogen-associated molecular patterns (PAMPs) that establish the initial barrier in the host to fend off pathogen establishment. The R genes enable an additional line of host defense if the pathogen can suppress the PTI response using their pathogenic effectors^42,43^. The R-gene expression was up-regulated in the resistant genotype ZC108 compared to the susceptible host, which may be significant for effector-triggered immunity (ETI). As soon as the R-genes are stimulated, downstream immune response participating in cellular reinforcement is triggered to thwart pathogen aggression^42,44^. Similar outcomes are also attained for MAPKs, which are ETI modulators, directly processing pathogen-induced signals from the receptors to downstream pathways.

Contrasting expression patterns in a plant immune-related gene set were observed in the susceptible and resistant background. The widely distributed plant defense proteins, Polygalacturonase-inhibiting proteins (PGIPs), are found in plant cell walls and prevent microbial invasion by inhibiting the activity of fungal polygalacturonases (PGs) activity. Chen et al.^45^ demonstrated that overexpressing the rice PGIP1 increases resistance to *Rhizoctonia solani* sheath blight. Similarly, PBL27/RLCK185 induced chitin-triggered immune responses against *Botrytis cinerea* in *Arabidopsis*^46^. NLRs can identify effectors when they are introduced into host cells during infection. PRR-triggered and NLR-triggered immunity (PTI and NTI) activate downstream defense reactions, including reactive oxygen species (ROS) production, extracellular calcium influx, map kinase activation, and transcriptional reprogramming for defense^47^. NB-LRR R proteins, such as RPM1, detect changes in RPM1-interacting protein 4 (RIN4) caused by AvrRpm1 and AvrRpt2 effectors, resulting in ETI. Immediately following pathogen infection, avrRpt2 and RPS2 induce AIG1 (avrRpt2 induced gene 1), which may cause cell death^48,49^. During fungal invasion, the WRKY33 gene is up-regulated, promoting transcription of defense-related genes such as camalexin biosynthetic genes. NRG and SAR proteins from the SA activating pathway and FMO1 from the pipecolic acid pathway are found to be down-regulated in resistant plants during infection, resulting in increased regulation of JA signaling and consequent defense responses^50,51^.

Plant biotic stress responses involve synthesizing and signaling through phytohormones like SA, JA, and ethylene^52^. The present analysis also agrees with this idea, as SA and JA-responsive genes in the pathways were significantly enriched in *L. theobromae* challenged tea plants at 1 DPI. However, in most predicted conditions, ethylene biosynthesis and signaling prevailed. According to a previous study, ethylene signaling and ethylene production were necessary for rice to have resistance against blast fungus *Magnaporthe oryzae*^53^. Ethylene-insensitive mutant lines were shown to have declined expression of chitin-binding receptors, PR proteins, phytoalexin-synthesizing enzymes, and reduced hypersensitive response. Thus, according to our observation, the involvement of ethylene-mediated pathways is considered critical for the resistance of host plants under fungal pathogen attack. Furthermore, elevated expression of the genes for the Cytochrome P450 monoxygenase and receptor kinase families were observed, pointing to their potential roles in conferring pathogen defense.

Following fungal inoculation, tea leaves are significantly enriched in DEGs important for transcriptional regulation. Though varied in number, MYB, ERF, bHLH, C2H2-ZF, C2C2, WRKY, and NAC family games were most dynamic in response to the studied seven diseases. The WRKY and NAC families are the source of most transcription factors (TFs) involved in pathogen defense mechanisms and responses^27,54,55^. These gene families contribute to plant immunity by differentially regulating defense-related gene expression. Most of the differentially expressed TFs, in response to the gray blight of tea, are from MYB, ERF, and NAC families^17^. Previous RNA-seq experiments showed that ERF positively regulates phytoalexin production and induces flavonoid biosynthesis pathway genes, thereby conferring pathogen resistance^56,57^. In an earlier study, components of the WRKY family were discovered to participate in biotic stress reactions, especially fungal infection in tea^58^. Moreover, earlier reports stated that NAC TFs regulate plant immunity to biotrophic, hemibiotrophic, or necrotrophic pathogens by modulating hypersensitive responses^59–61^. Present meta-analyses driven hub genes also comprised at least one member of ERF, bZIP, MYB, bHLH, C2H2-ZF transcription factor families, deciphering their crucial role in the gene regulation under biotic stress. Other transcription factor families are also involved in rapid transcriptional reprogramming of downstream genes, which contributes to fine-tuning immune responses.

Secondary metabolite production in response to pathogenic ingression is an additional essential element of host defense. Multiple transcripts involved in the biosynthesis of secondary metabolites, including flavonoids, isoprenoids, and phenylpropanoids, were mapped by pathway enrichment analysis. These substances act as building blocks for synthesizing antimicrobial compounds, which perform crucial roles in the immune response against pathogens^62,63^. In addition, the secondary metabolites produced by tea plants have a major impact on the flavor and quality of tea^64,65^. The present study revealed that the regulation of key genes that help the biosynthesis of secondary metabolites, particularly the phenylpropanoid pathway, was differentially expressed in the seven fungal stresses. These genes were mostly up-regulated on infection except in the resistant genotype, which showed a strong down-regulation of these flavonoid biosynthetic genes at the early stages of infection. Earlier reports state that the sensitive variety has greater levels of flavonoid accumulation than the highly resistant ones^66–68^. According to Nisha et al. ^69^, resistant and susceptible cultivars of tea differ in how they regulate the genes involved in the development of the tea flavonoid pathway. Anthocyanidin reductase (ANR), which catalyzes the production of tea epicatechin or epigallocatechin, is enhanced in susceptible individuals during the early infection stage^69^. Supporting evidence from the current analysis points to the specialized metabolites' role as host arsenals when pathogen ingression has already occurred rather than their activity in the immune genotypes toward an incompatible environment for the pathogen.

Our GO enrichment results are comparable to other studies demonstrating that various functional groups of DEGs under biotic stress were related to metabolic pathways, regulatory mechanisms, and stimulus-responsive pathways^25,27^. During the host–pathogen interaction, "response to stimulus" was a prominent category that remained enriched with defense-related genes. On the other hand, a common plant response to the majority of pathogens is reduction–oxidation (redox) signaling^70^, which has been found to have significantly enriched GO terms among the consensus DEGs. Some redox-related genes, such as catalases and NADPH oxidases, were necessary for immunity, indicating that the redox state might further regulate plant defense responses^71^. The significance of reactive oxygen species (ROS) in tea plants was noted concerning the potential activation of a defense mechanism against the destructive fungal pathogen *Exobasidium vexans*^67^. Accordingly, present findings support the fact that during defense, multiple ROS signals are combined^72^. However, due to the significant induction of free radicals, the host cells may experience oxidative stress. Therefore, at various phases of fungal pathogenesis in tea, detoxification enzymes (i.e. glutathione S transferase, peroxidases etc.) maintained a steady-state level of free radicals, which may underlie a robust immune response.

Signatures in KEGG pathway based GSEA demonstrated a steady modulation of the MAPK signaling pathway. It is essential in triggering the host response by transducing diverse extracellular stimuli and activating pertinent regulatory cascades during fungal pathogenesis. The enrichment analysis also showed the extent of plant metabolic responses, including phenylpropanoid biosynthesis, when challenged with fungal pathogens. Pathogens or pathogen-derived elicitors change the metabolism of carbohydrates, proteins, and lipids as sugars and amino acids are intermediates in the pathways that generate specific defense metabolites^27,73–75^. Phenylpropanoids and flavonoids are plant defense metabolites attributed to prevent invasion or serve as toxic weapons against microbial targets^76,77^. The DEGs were subjected to co-expression network analysis to identify the processes and mechanisms contributing to the fungal stress response. Most of the hub genes found in this study were connected to the organization of the plant cell wall, a dynamic barrier encountered by many pathogens. These include cutin synthases, which facilitate the production of cutin and cuticular waxes, a protective layer covering the aerial surface of plants. This hydrophobic layer plays a pivotal role during biotic stress by reducing non-stomatal water loss^78^. Other important factors identified in the category include the xylan biosynthesis enzyme, pectin methylesterase, pectate lyase, expansins, etc. All these are reported to be conspicuously involved in plant cell wall-mediated immunity^79^.

MicroRNAs, a large class of non-coding RNAs, are involved in post-transcriptional gene regulation, associated with stress responses^28^. The present study explored miR169, miR399, miR156, miR171 and miR172 members, which are reported to regulate DEGs involved in PTI and ETI^80^. Zhang et al.^81^ found that miR156 and miR395 suppressed WRKY transcription factors, reducing PR gene expression and consequently influencing tolerance to the apple leaf spot fungus *Alternaria alternata* f. sp. *mali*. The predicted miRNA-DEGs pairs demonstrated that WRKY, GRAS, and SBP transcription factors are targets of one or more miRNAs. Also, some important components of the ETI network as well as the identified hub gene (CSS0007273) belonging to the flavonoid biosynthesis pathway, were found to be regulatory targets of miRNA-mediated switches. It was comparable to the previous report of small RNA sequencing based identification of differentially expressed miRNAs and their targets in tea^17^. In both contexts, miR397 targeted laccase family genes, further confirmed in the degradome-supported conserved miRNA target database. Similarly, miR530 targets hydrolases encoding transcripts. The present findings shed light on the essential miRNAs that act as a regulatory repertoire to manifest the fungus-induced responses in tea plants. Nevertheless, the differential expression of transcripts was not always solely dependent on the miRNA-mediated degradation route, as there is other post-transcriptional mode of regulation to act, such as differential alternative splicing and nonsense-mediated decay^82,83^.

Physical mapping of the common fungal-responsive genes revealed that they were primarily found on the clusters at the upper and lower ends of the tea chromosomes. Most DEGs were duplicated in the tea genome with their syntenic pairs in other chromosomes. Throughout evolution, flowering plants underwent several whole genome duplications and subsequent rounds of genome fractionation^84^. The paralogous repeats that followed the whole-genome duplication event significantly affected the copy number of necessary genes^65^. Due to the overrepresentation of syntenic genes among DEGs, it was suggestive that the paralogous are more prone to transcriptional adjustment^84^. Specific expansion of the core stress-responsive genes arose through tea plant evolution, resulting in transcriptionally active multi-copy gene families. These findings will serve as valuable resources to identify disease resistance and quality traits associated QTLs in tea, an understudied area until now.

## Materials and methods

### Retrieval of genome and transcriptome datasets

Tea genome sequence at chromosome level^22^ was extracted from the Tea Plant Genome Database^85^ (http://eplant.njau.edu.cn/tea/). Gene ontology (GO) information of the reference genome was downloaded from the same portal. A total of 102 RNA-seq datasets were harvested from the NCBI-SRA, comprising the raw sequence reads of mRNAs originating from tea leaves uninfected and infected groups of seven fungal pathogens viz. *C. camelliae* (BioProject: PRJNA396805)^14^, *D. bellidis* (BioProject: PRJNA800776), *D. segeticola* (BioProject: PRJNA528172)^18^, *E. sorghinum* (BioProject: PRJNA799860)^19^, *L. theobromae* (BioProject: PRJNA752965)^16^ *P. trachicarpicola* (BioProject: PRJNA756832)^13^ and *Pseudopestalotiopsis* sp. (BioProject: PRJNA564655)^17^.

### Reference-based transcriptome assembly

The FastQC tool v 0.11.9^86^ was utilized to determine the quality of the raw RNA-seq datasets. Trimmomatic v 0.38^87^ was used to eliminate adapter nucleotide sequences and low-quality reads from both ends and across the entire read. HISAT2^88^ was utilized to map trimmed RNA-seq reads against the chromosome-scale reference genome sequence of tea^22^. StringTie v 2.1.7^89^ was used to assemble and quantify the transcriptome from the aligned BAM files of the mapped RNA-seq reads.

### Analysis of differential gene expression

Using StringTie, expression values were calculated and normalized as TPM (Transcript per kilobase Million), and transcripts with TPM value 1 were considered expressed and used for further analysis. The fungal infection-responsive DEGs was measured using DESeq2^31^, which provides statistical methods for identifying DEGs based on a negative binomial distribution model. The batch effects among datasets were corrected using the ComBat^90^ tool in R, and the effectiveness of the batch factor correction was assessed using principal component analysis (PCA) both before and after normalization. Transcripts with a |log2foldchange| value of 1 and above with a false discovery rate (FDR) adjusted p-value (p-adj) of 0.05 were considered differentially expressed.

### Functional enrichments analyses

GO enrichment of the identified consensus DEGs was performed using ‘goseq’ package^91^ in R. Significantly enriched GO terms with FDR corrected p < 0.05 were categorized according to their respective components, i.e. CC, BP, and MF. Over-represented GO categories were represented as bubble plot using ‘ggplot2’, ‘dplyr’, ‘hrbrthemes’ ‘viridis’ packages in R to generate the enrichment profiles. KEGG pathway based gene set enrichment analyses were undertaken using ExpressAnalyst^92^ platform that implements ‘fgsea’ R package^93^ to find out the association of DEGs in corresponding pathways. The Mercator tool (https://www.plabipd.de/portal/web/guest/mercator-sequence-annotation)^94^ was used to perform MapMan annotation of the identified DEGs. MapMan was used to visualise the expressional fold changes of the identified DEGs in each condition according to DESeq2 output were visualised in MapMan application^32^. Enrichment analyses within the MapMan pathways were carried out through The Wilcoxon Rank Sum Test with Benjamini–Hochberg correction of the obtained p-values. To identify the possible interactions among the core set of DEGs, an analysis of protein–protein interactions (PPI) network was carried out using *Arabidopsis* as a reference in STRING database.

### Identification of potential miRNAs targeting candidate genes

The miRNA-mediated post-transcriptional gene regulation was assessed utilizing the psRNATarget server^35^ (http://plantgrn.noble.org/psRNATarget/) to identify the miRNA target sites exist within the identified DEGs. This server is utilized to find out the possible targets of small RNAs by estimating complementarity between them using a scoring scheme implementing the unpaired energy (UPE). The mature miRNA sequences of tea plant, harvested from PmiREN^95^ database, were served as query against the pool of differentially expressed transcripts. Significant miRNA-target pairs were investigated for confirmation with the degrodome-supported, experimentally validated miRNA target reports available in the TarDB^36^ portal.

### Chromosomal distribution and synteny analyses

The chromosome locations of the fungal infection-responsive core set of DEGs were excavated from the reference genome annotation file. Chromosomal location map was drawn using the using SRPlot (https://www.bioinformatics.com.cn/en). For the synteny analysis within the genome, the reference proteome sequences were used to compare the homology. The MCScanX^96^ program was used to investigate syntenic relationships and collinearity between the genes. The identified meta-DEGs were represented along with their syntenic pairs in a Circos^97^ diagram.

### Co-expression network analyses

The matrix of normalized expression values of DEGs for all samples was subjected to a weighted gene co-expression network analysis (WGCNA)^98^ in order to identify groups of DEGs with similar expression patterns. The following parameters were used to create a gene expression adjacency matrix for analysing the network topology: soft threshold power, 12; TOMType, unsigned; minModuleSize, 30. The scale free topology model and mean connectivity plots indicated the basis of selection of this soft threshold. To pinpoint gene co-expression modules, a dynamic tree-cut algorithm was used. The networks of gene co-expression were visualized using Cytoscape v.3.10.0^99^. The genes with the highest connectivity in a module were identified as hub genes implicated by cytoHubba^100^.

### Ethical approval

All studies on plants complied with relevant institutional, national, and international guidelines and legislation.

### Supplementary Information

Supplementary Figures.

Supplementary Tables.

## Data Availability

The datasets analysed during the current study are available in the NCBI-SRA (https://www.ncbi.nlm.nih.gov/sra) repository under BioProject Accession numbers—PRJNA396805, PRJNA752965, PRJNA756832, PRJNA564655, PRJNA800776, PRJNA528172 and PRJNA799860.
