# Supplementary material for: Global transcriptome analysis reveals fungal disease responsive core gene regulatory landscape in tea
Source: Sci Rep. 2023 Oct 11;13:17186. doi: 10.1038/s41598-023-44163-x (PMC10567763; doi:10.1038/s41598-023-44163-x)
Supplement: Supplementary file 1 — Supplementary Figures. [file 41598_2023_44163_MOESM1_ESM.doc]

**Supplementary Information**

**Global transcriptome analysis reveals fungal disease responsive core gene regulatory landscape in tea plant**

Anjan Hazra^1^, Sanatan Ghosh^1^, Sudipta Naskar^1^, Piya Rahaman^1^, Chitralekha Roy^1^, Anirban Kundu^2^, Rituparna Kundu Chaudhuri^3^, Dipankar Chakraborti^1*^

^1^Department of Genetics, University of Calcutta, 35, Ballygunge Circular Road, Kolkata, 700019, India

^2^Plant Genomics and Bioinformatics Laboratory, P.G. Department of Botany, Ramakrishna Mission Vivekananda Centenary College (Autonomous), Rahara, Kolkata, 700118, India

^3^Department of Botany, Barasat Govt. College, 10, KNC Road, Barasat, Kolkata, West Bengal, India

*Correspondence: [dcgntcs@caluniv.ac.in](mailto:dcgntcs@caluniv.ac.in)


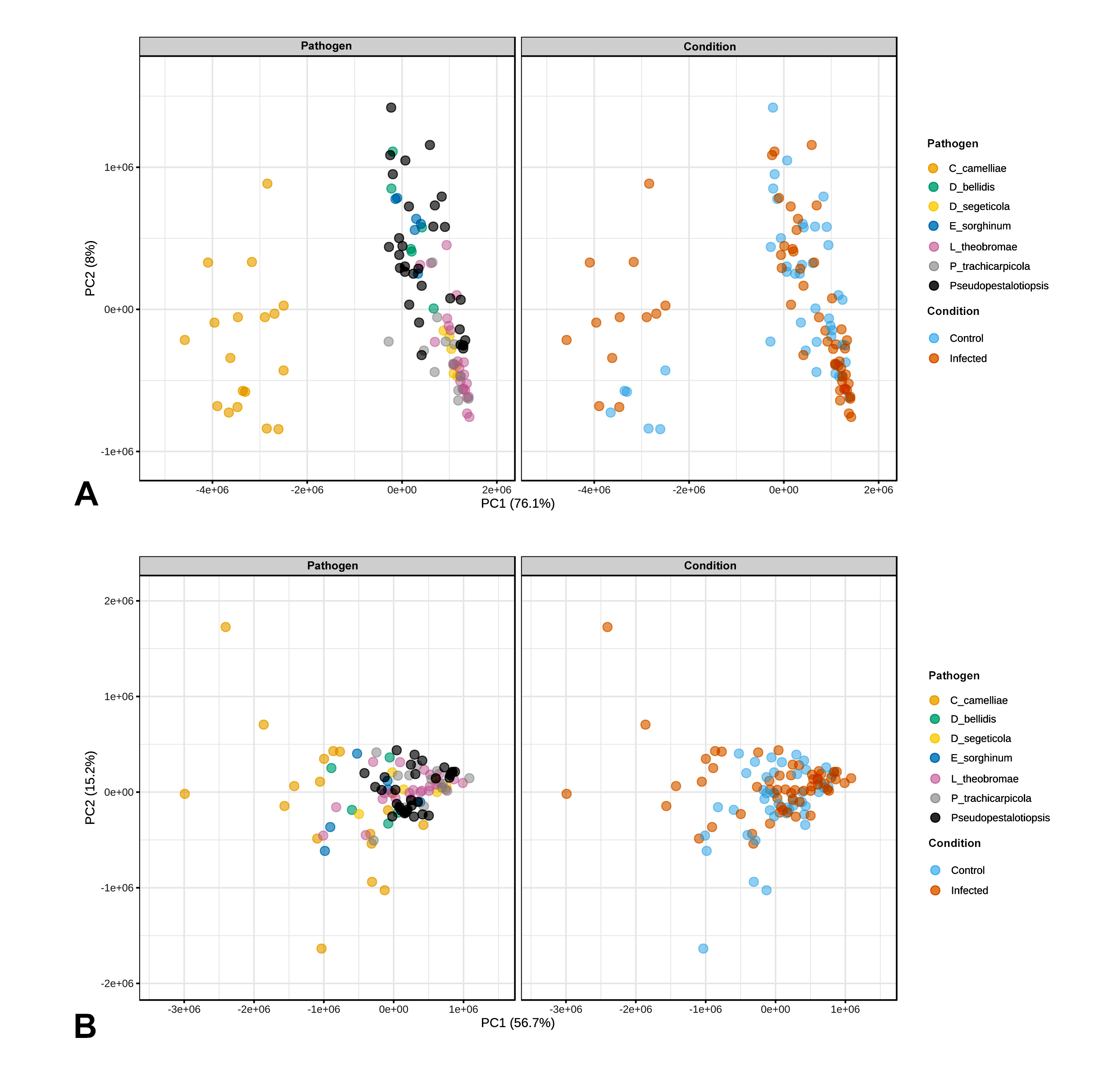


Supplementary Figure S1: PCA plots of normalisation of the batch effect. Samples are grouped by gene expression profiling, both before (A) and after (B) batch correction on raw data.


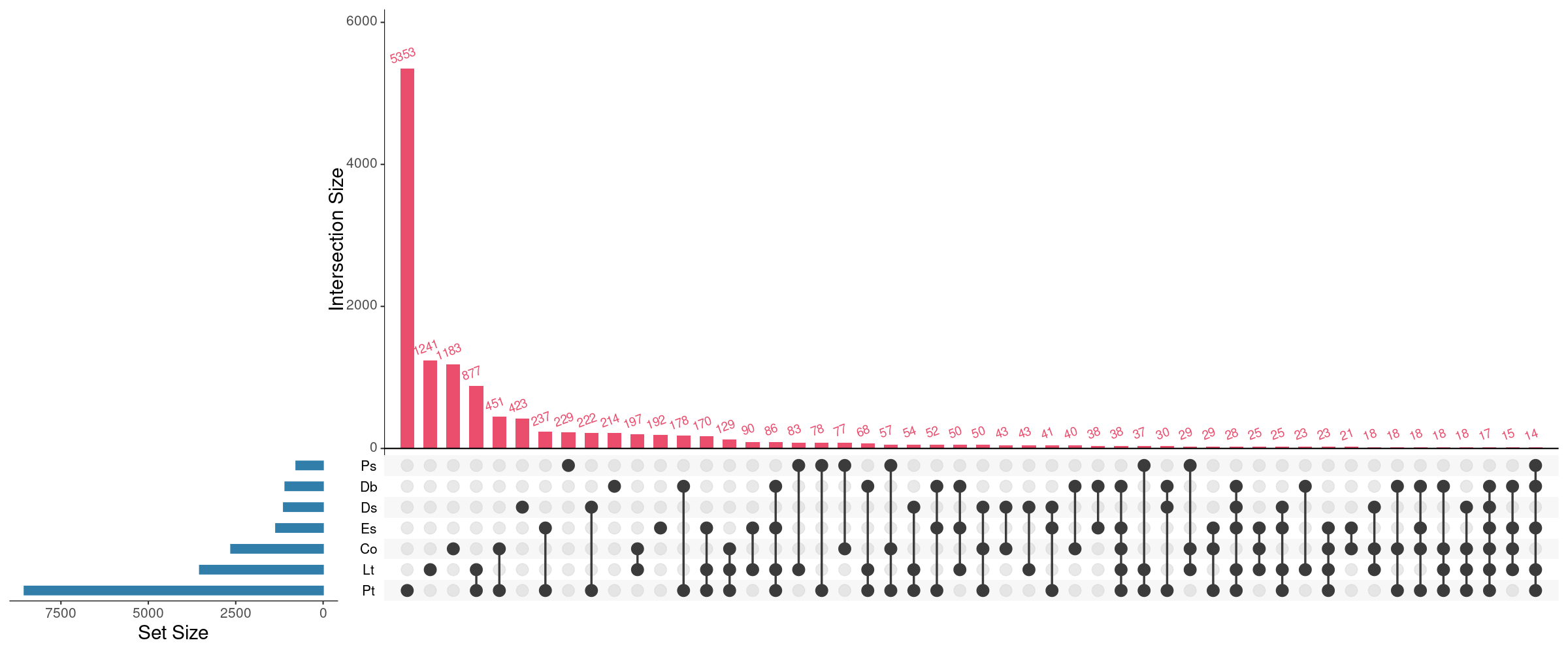


Supplementary Figure S2: Comparison among observed DEGs observed in tea plant against all studied pathogen infection determined using an upset plot.


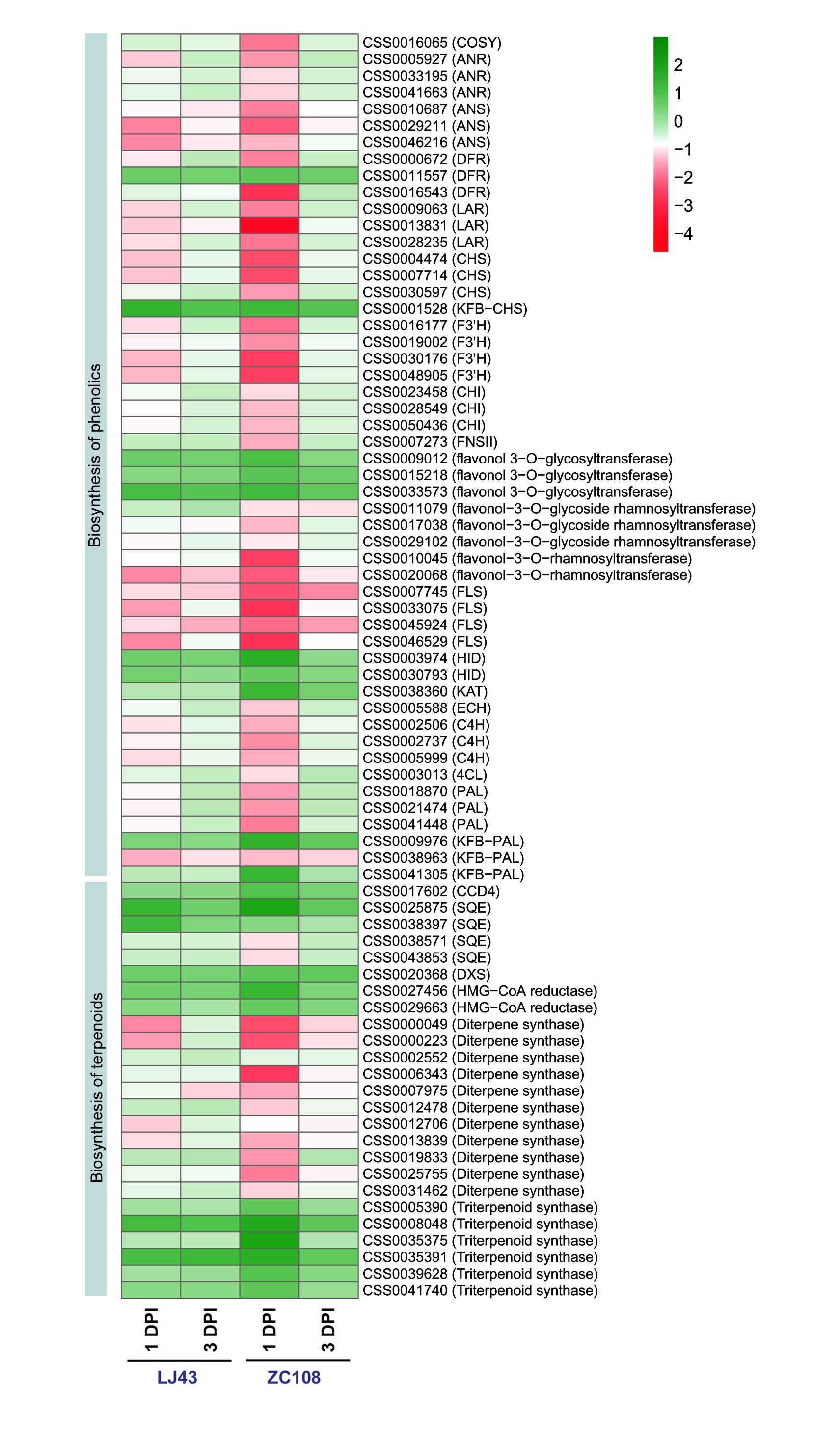


Supplementary Figure S3: Heatmap depicting expressional fold change of secondary metabolism pathway genes between susceptible and resistant genotype. ANR, anthocyanidin reductase; ANS, anthocyanidin synthase; 4CL, p-coumarate:CoA ligase; C4H, cinnamate 4-hydroxylase; CCD4, class-CCD4 carotenoid cleavage dioxygenase; CHI, chalcone isomerase; CHS, chalcone synthase; COSY, coumarin synthase; DFR, dihydroflavonol 4-reductase; DXS, 1-deoxy-D-xylulose 5-phosphate synthase; ECH, enoyl-CoA hydratase; F3'H, flavanone 3-hydroxylase; FLS, flavonol synthase; FNSII, type-II flavone synthase; HID, 2-hydroxyisoflavanone dehydratase; KAT, 3-ketoacyl-CoA thiolase; KFB-CHS, regulatory substrate adaptor of SCF ubiquitin ligase; KFB-PAL, substrate adaptor of regulatory SCF ubiquitin ligase; LAR, leucoanthocyanidin reductase; PAL, phenylalanine ammonia lyase; SQE, squalene epoxidase.

**
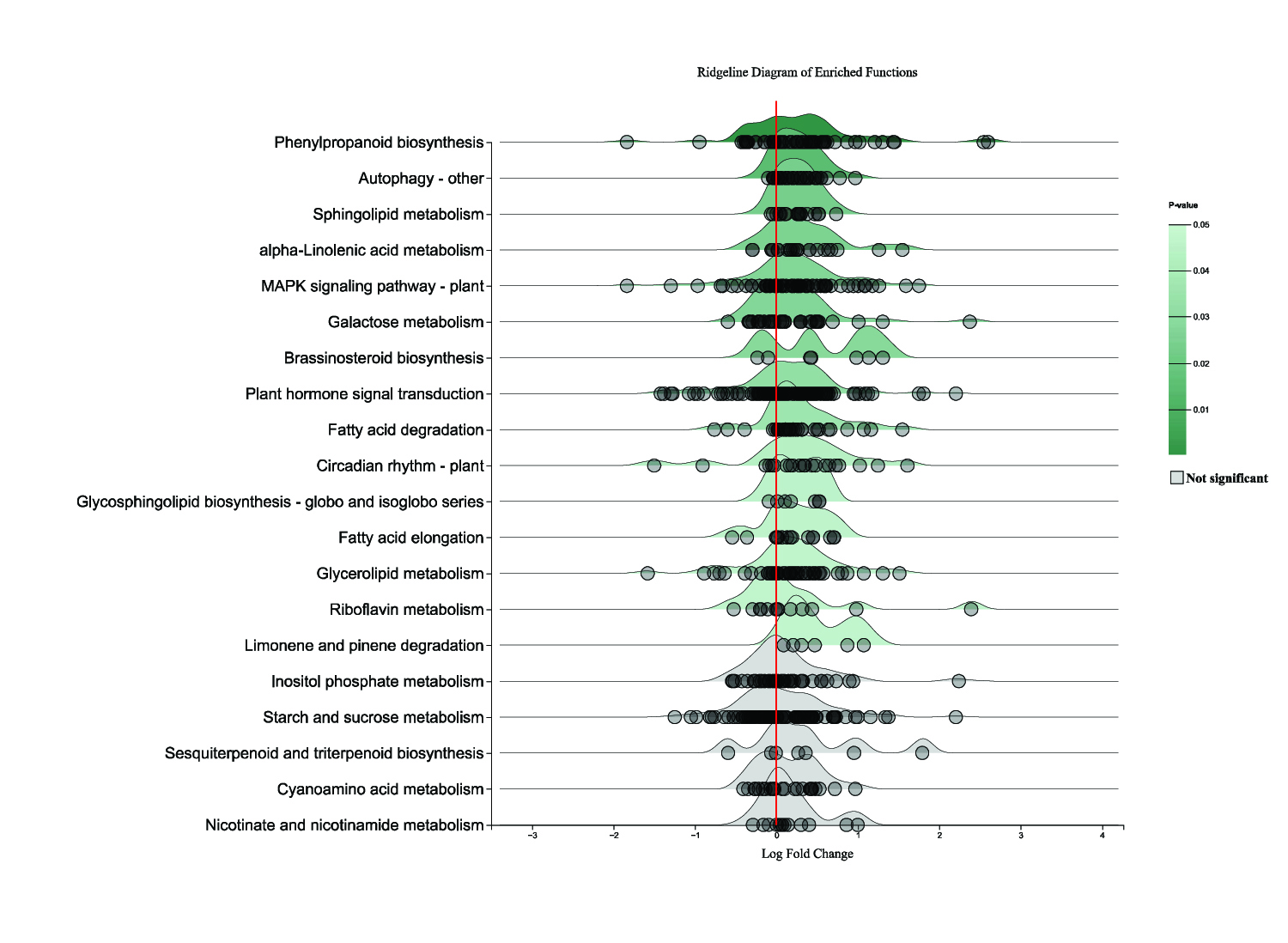
**

Supplementary Figure S4: Ridgeline plots displaying enrichment of DEGs upon treatment of *Colletotrichum camelliae*

**
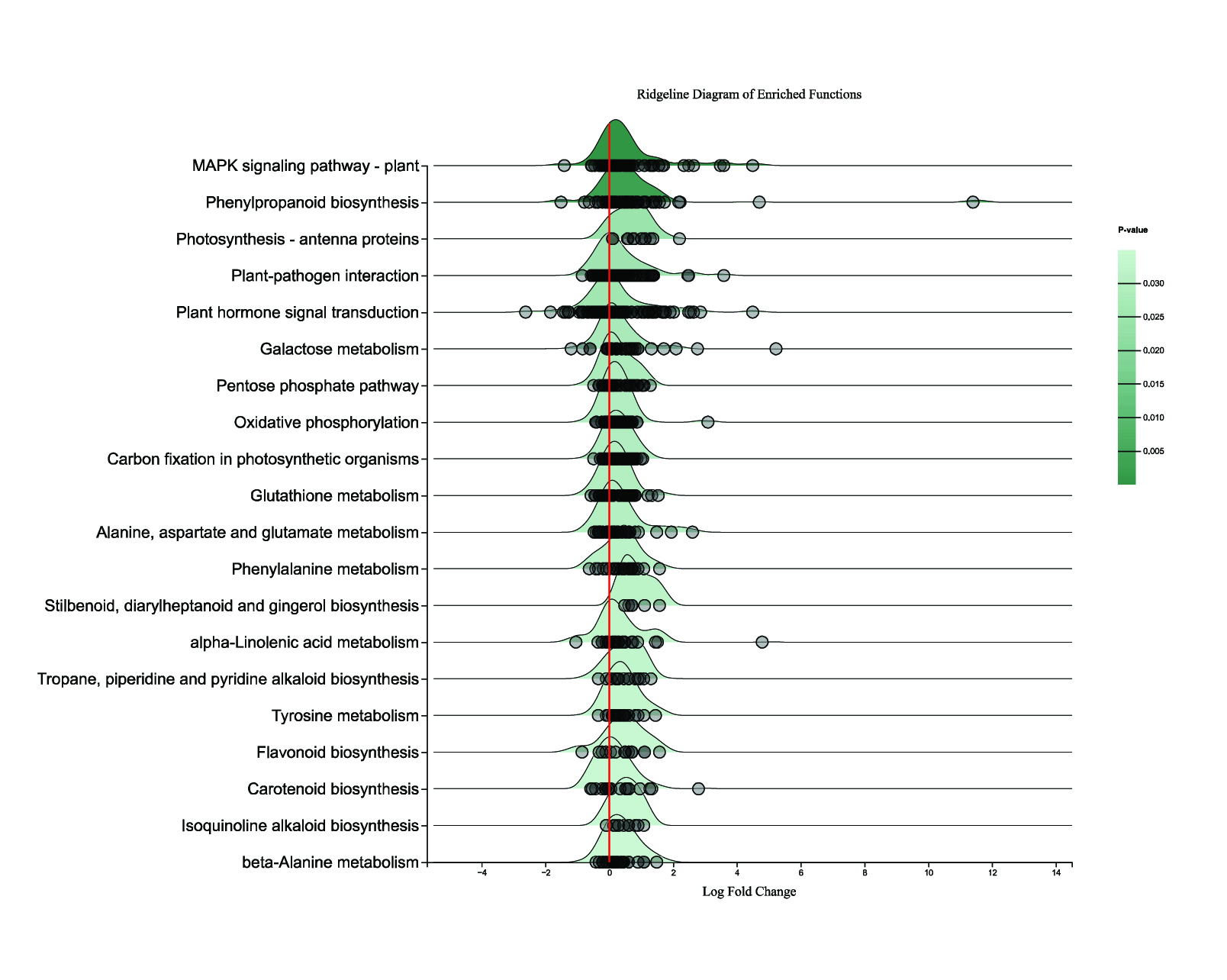
**

Supplementary Figure S5: Ridgeline plots displaying enrichment of DEGs upon treatment of *Didymella bellidis*

**
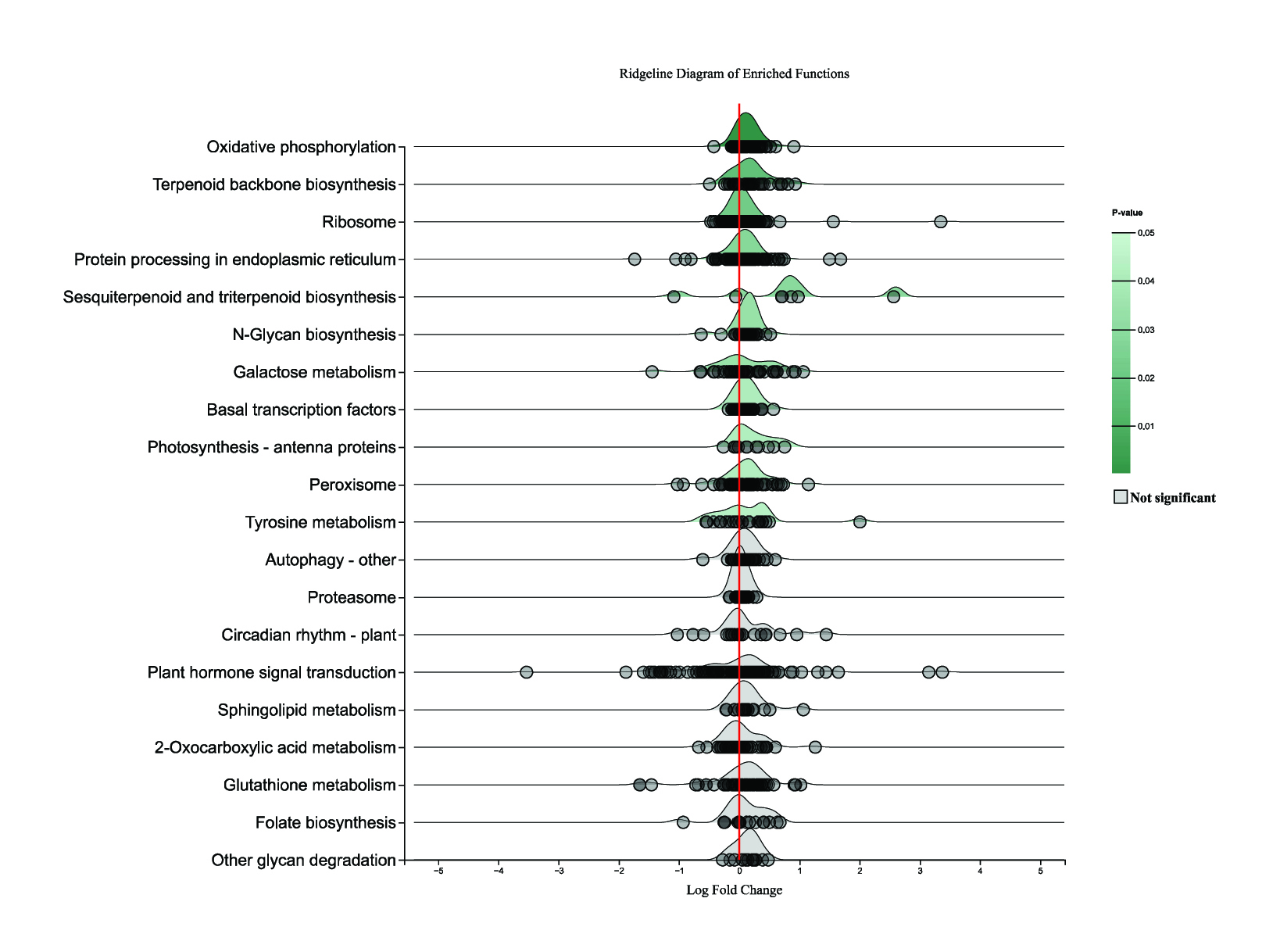
**

Supplementary Figure S6: Ridgeline plots displaying enrichment of DEGs upon treatment of *Didymella segeticola*

**
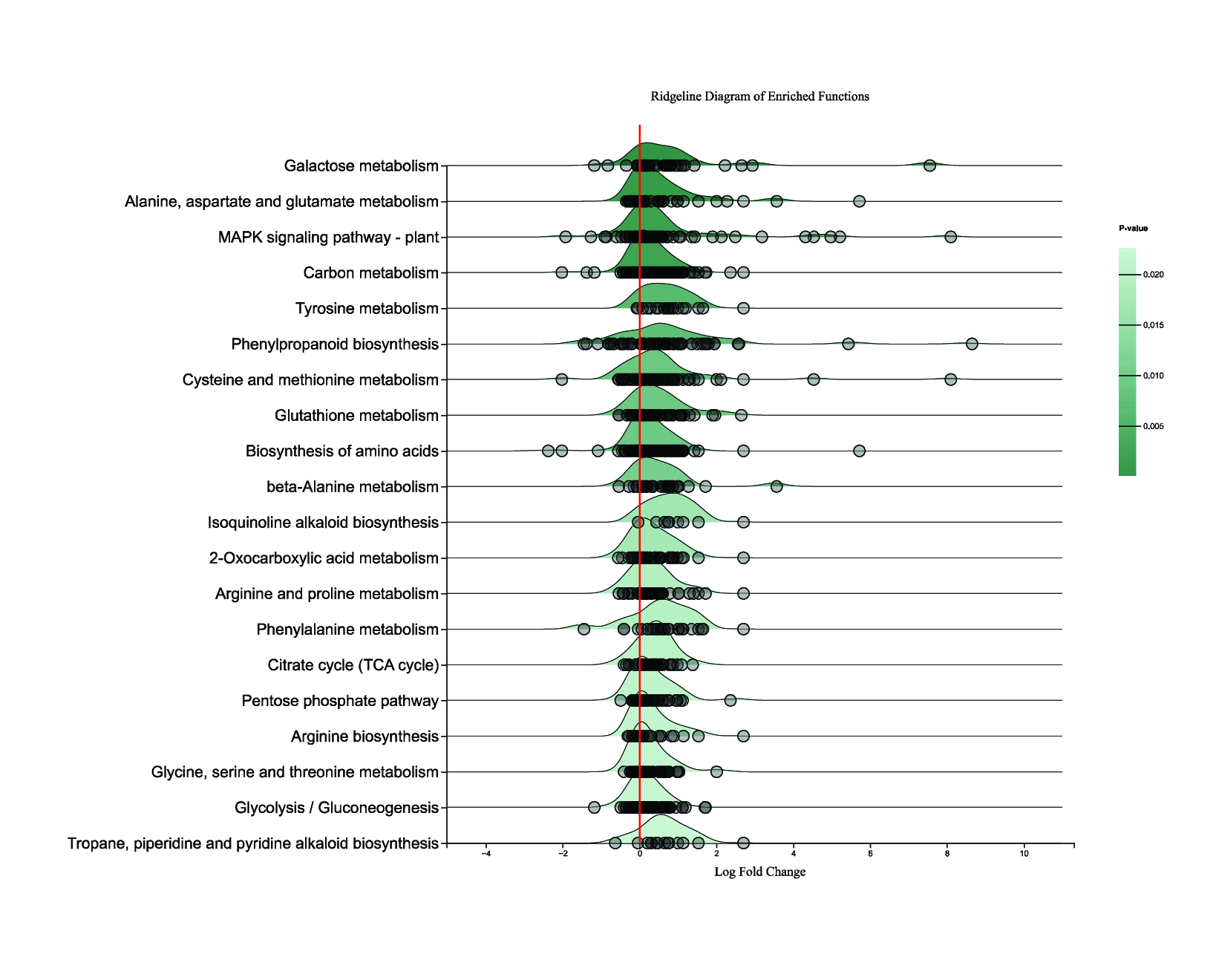
**

Supplementary Figure S7: Ridgeline plots displaying enrichment of DEGs upon treatment of *Epicoccum sorghinum*

**
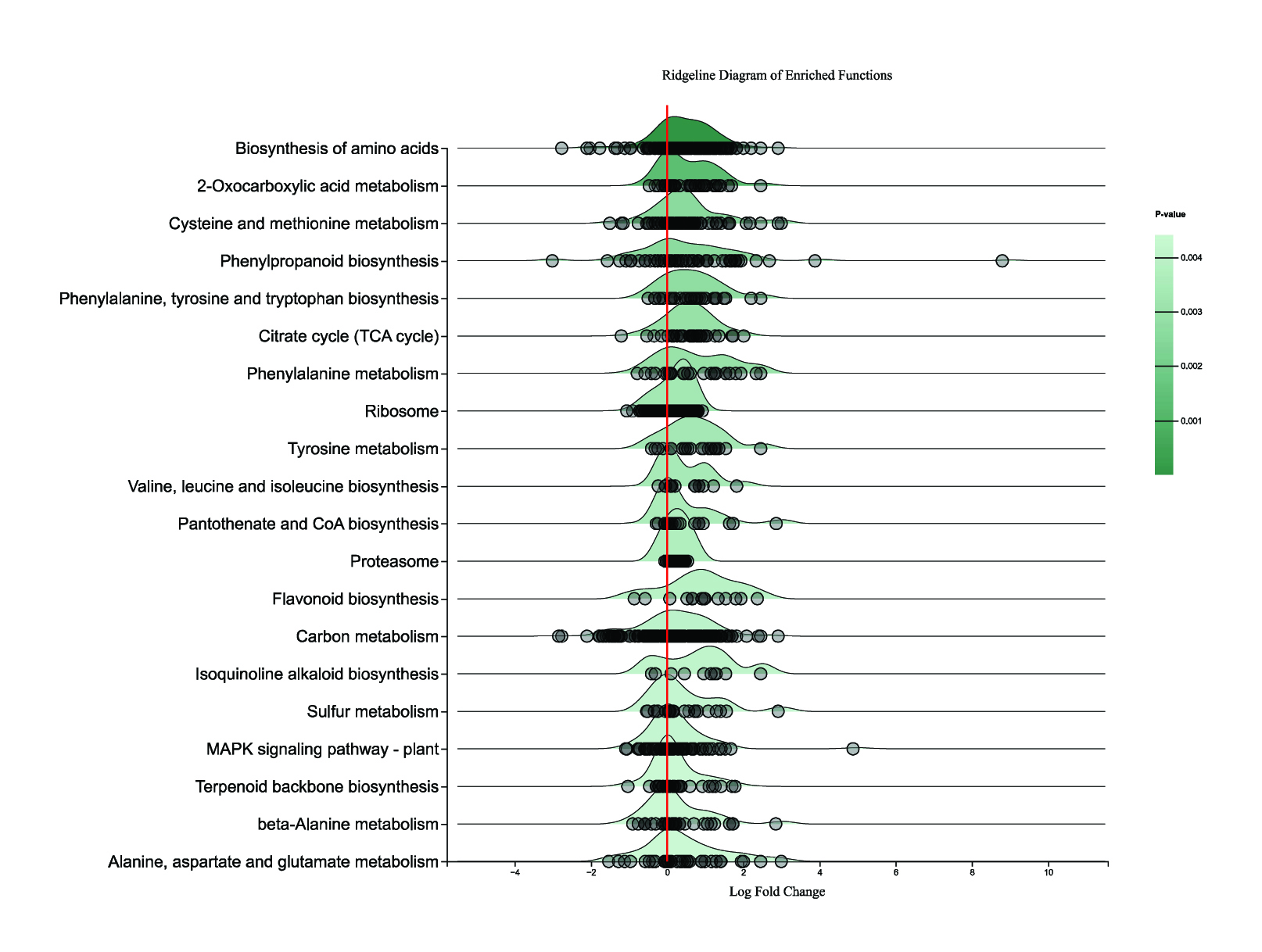
**

Supplementary Figure S8: Ridgeline plots displaying enrichment of DEGs upon treatment of *Lasiodiplodia theobromae*

**
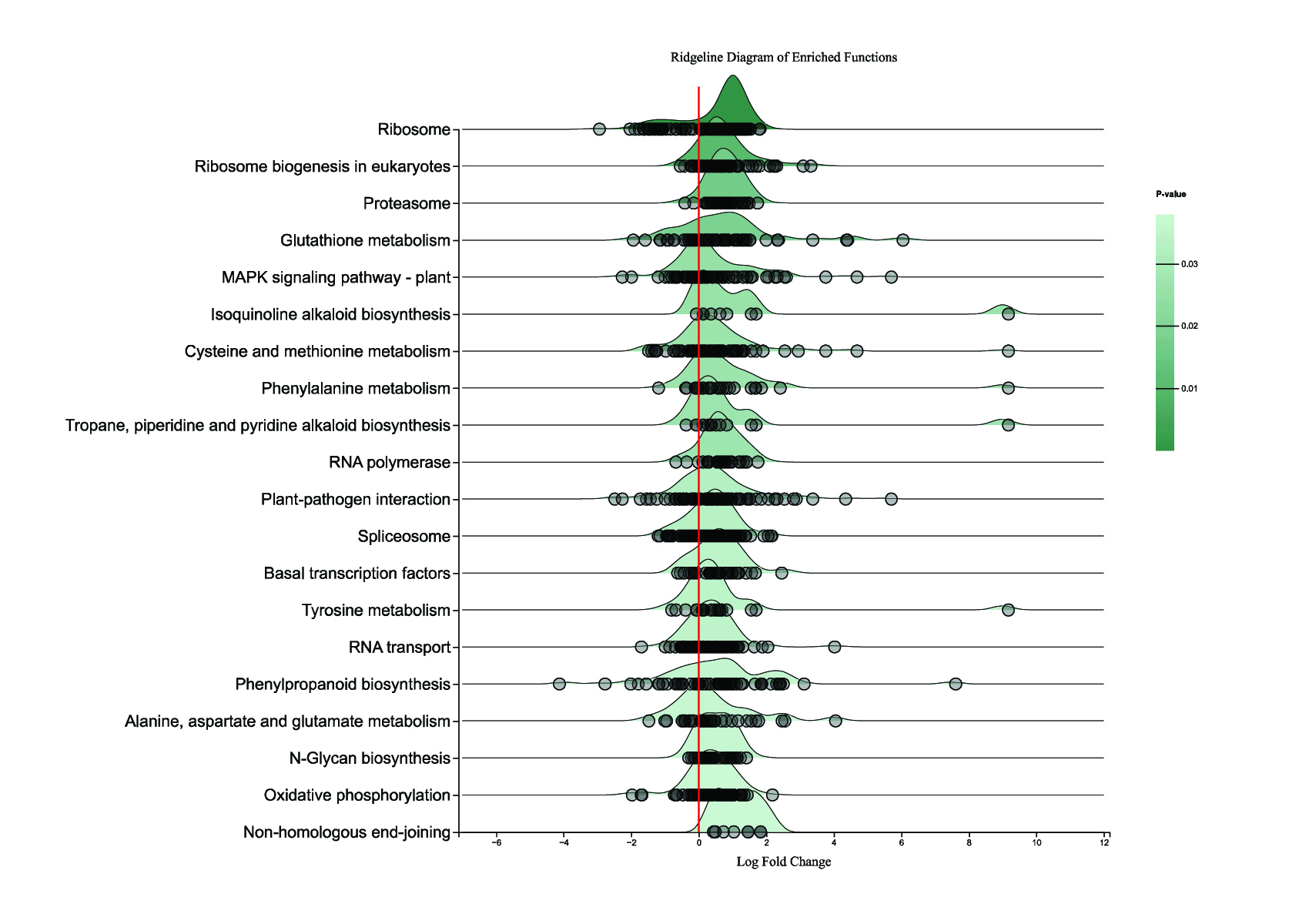
**

Supplementary Figure S9: Ridgeline plots displaying enrichment of DEGs upon treatment of *Pestalotiopsis trachicarpicola*


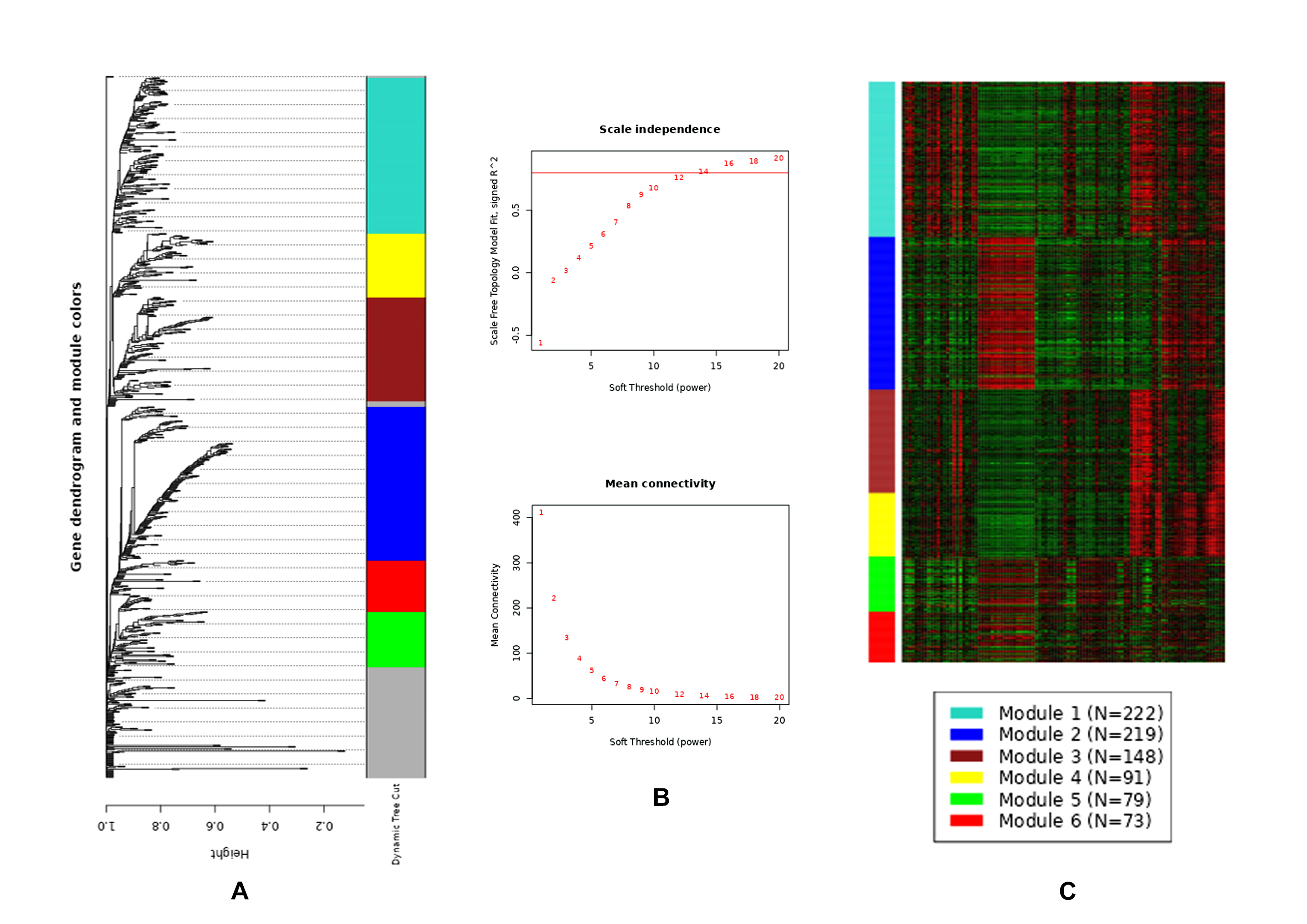


Supplementary Figure S10: Weighted gene co-expression network analysis (WGCNA) of the meta-DEGs in individual conditions. Hierarchical cluster tree with the color bands representing the assigned modules (A), scale-free fit index of the network topology calculated using a soft-thresholding power analysis, heatmap showing the expressional dynamics of modules in studied samples (C).
